# Comprehensive Analytical Investigation of the Photodegradation of Olopatadine and In Silico Toxicity Assessment of Its Photodegradation Products

**DOI:** 10.3390/molecules31173112

**Published:** 2026-09-04

**Authors:** Anna Gumieniczek, Dominika Buś, Ewa Oleszek, Beata Naumczuk, Piotr Hołowiński

**Affiliations:** 1 Department of Medicinal Chemistry, Faculty of Pharmacy, Medical University of Lublin, Jaczewskiego 4, 20-090 Lublin, Poland; dominika.bus29@gmail.com (D.B.); 61870@umlub.edu.pl (E.O.); 2 Institute of Organic Chemistry, Polish Academy of Sciences, Kasprzaka 44, 01-224 Warsaw, Poland; beata.naumczuk@icho.edu.pl; 3 Department of Chromatographic Methods, Faculty of Chemistry, Maria Curie-Sklodowska University in Lublin, Pl. Marii Curie-Sklodowskiej 3, 20-031 Lublin, Poland; piotr.holowinski@umcs.pl

**Keywords:** olopatadine, photodegradation, degradation products, LC-UV, UHPLC-HRMS/MS, NMR spectroscopy, in silico toxicity

## Abstract

Olopatadine (OLO) is a second-generation antihistamine approved for the treatment of allergic conjunctivitis as ophthalmic and nasal solutions. Due to its intrinsic absorption in the near-UV region (approximately 300 nm), OLO may be susceptible to photodegradation and understanding this behavior is essential for ensuring the quality and safety of OLO-containing pharmaceutical formulations. The forced photodegradation of OLO was investigated under UV/Vis irradiation (300–800 nm) over a wide pH range. Photodegradation kinetics was evaluated using a selective LC-UV method. OLO degradation followed first-order kinetics, with rate constants ranging from 3.45 × 10^−5^ to 6.91 × 10^−5^ s^−1^, corresponding to degradation levels in the range 45.54–81.95%. Photodegradation products were characterized using UHPLC-HRMS/MS, leading to the identification of twelve, including seven previously unreported compounds. Seven degradants were isolated by preparative LC-UV and their structures were confirmed by NMR spectroscopy, including three newly reported compounds. The potential toxicity of all identified photodegradants was evaluated using the in silico tools OSIRIS Property Explorer and Toxtree. Five products were predicted to exhibit reproductive toxicity and irritation potential, whereas one compound showed a potential tumorigenic risk. Overall, this study provides comprehensive insight into the photostability of OLO and the formation of its photodegradation products.

## 1. Introduction

**Figure 1:**
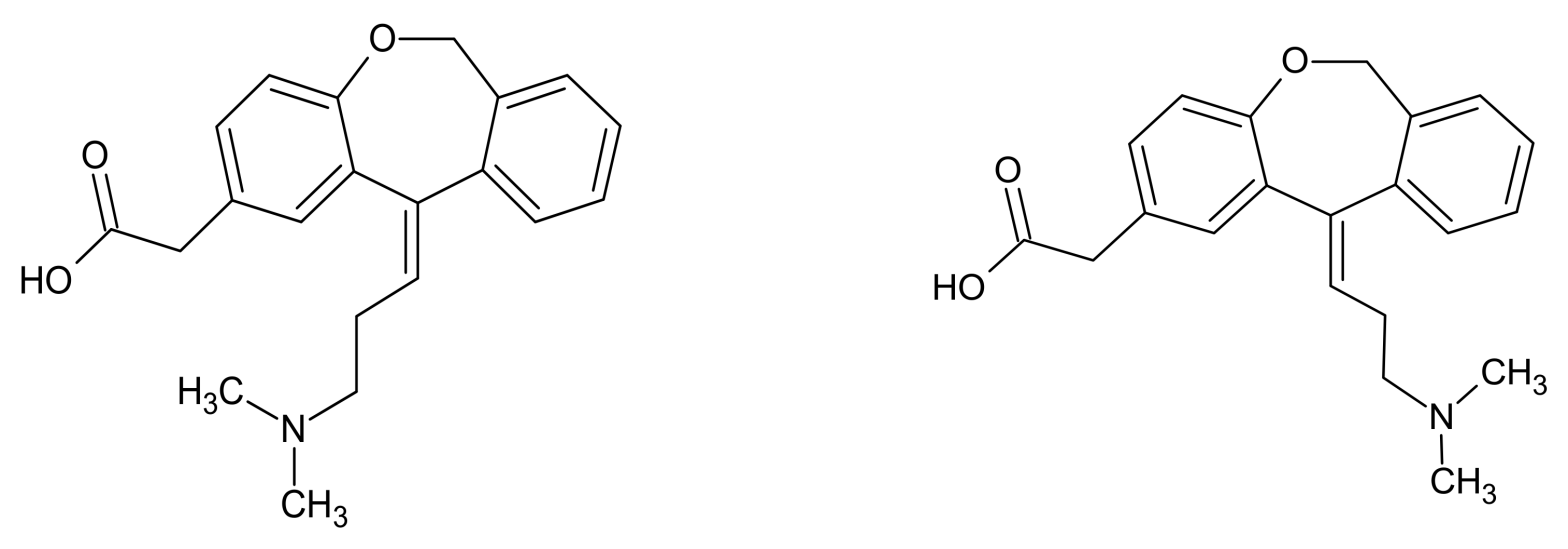
Chemical structure of olopatadine (OLO, Z-isomer) and its E-isomer.

Olopatadine (OLO) is a second-generation antihistamine approved by the U.S. Food and Drug Administration (FDA) in 1996 and the European Medicines Agency (EMA) in 2002 for the treatment of allergic conjunctivitis. It is a non-sedating histamine H1 receptor antagonist that also exhibits mast cell-stabilizing activity. This dual mechanism of action enables effective control of the signs and symptoms of seasonal allergic conjunctivitis and allergic rhinitis using ophthalmic and nasal formulations [1]. Structurally, OLO is a dibenzo[b,e]oxepine derivative containing a (dimethylamino)propylidene substituent attached to the oxepine ring and an acetic acid side chain on one of the aromatic rings of the tricyclic scaffold [1,2]. The pharmacologically active form of OLO is the Z-isomer (cis configuration), which is responsible for its antihistaminic and mast cell-stabilizing properties. Although both the Z- and E-isomers (Figure 1) exhibit comparable affinity for the H1 receptor, only the Z-isomer is marketed as the active pharmaceutical ingredient (API). The E-isomer is an undesired by-product formed during the stereoselective Wittig olefination used in OLO synthesis. Consequently, pharmaceutical laboratories use the E-isomer as a reference standard for the development and validation of analytical methods to detect trace levels of this impurity in olopatadine drug substance [3].

**Figure 2:**
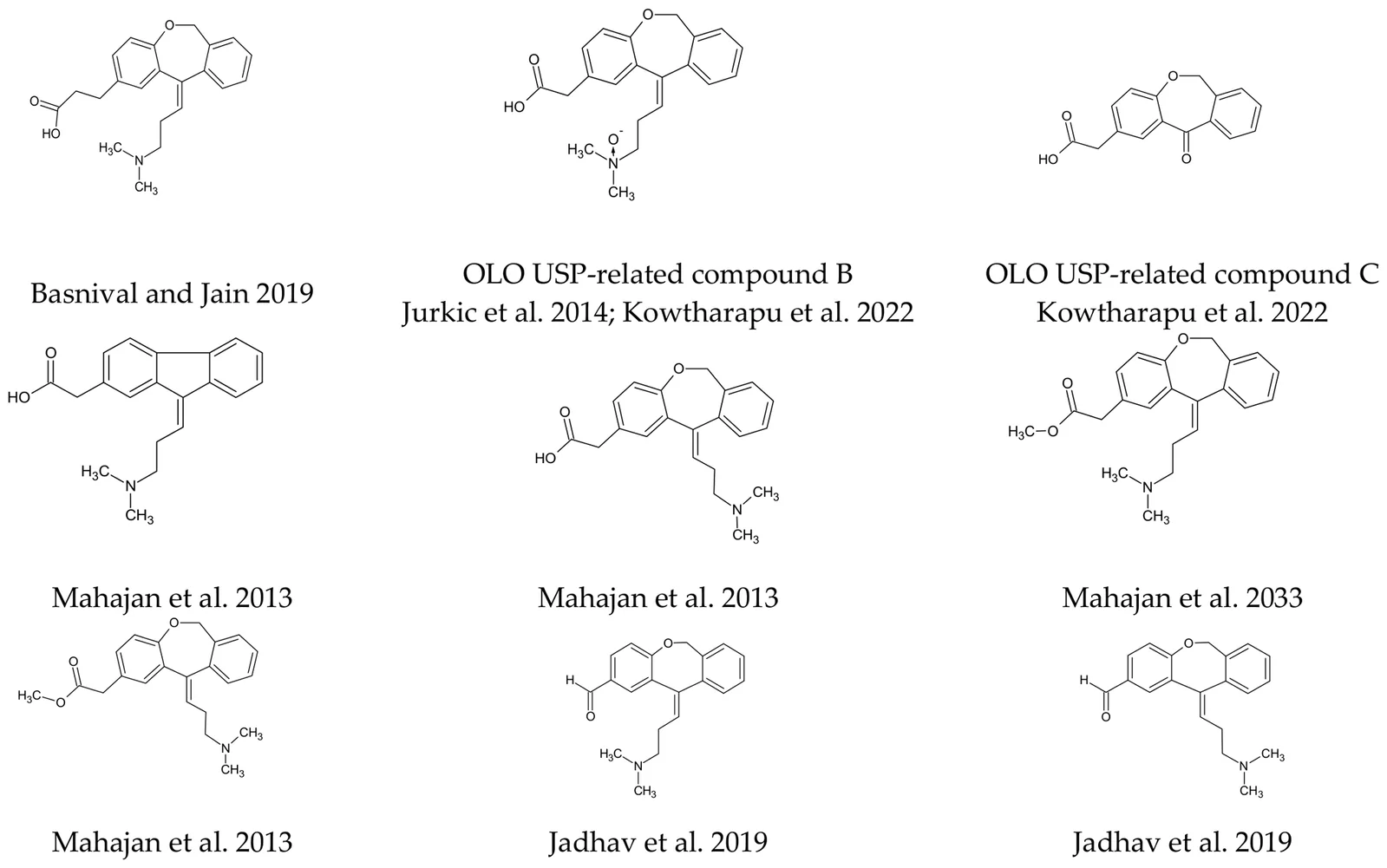
Photodegradation products of olopatadine (OLO) described in the literature [10,11,12,13,14].

Photodegradation encompasses a series of physicochemical changes initiated by light. When API absorbs light, it may undergo photochemical degradation, resulting in chemical transformations such as oxidation, isomerization, dimerization and the formation of photodegradation products. Consequently, photodegradation can lead not only to a loss of therapeutic activity but also to the formation of potentially harmful compounds that may increase the risk of adverse effects in patients [4,5,6]. According to the ICH Q1B guidelines, photostability testing should be performed for drug substances that absorb light at wavelengths above 290 nm [7]. However, routine photostability studies are often insufficient to detect all degradation products and comprehensively evaluate their toxicity. Therefore, forced photodegradation studies conducted under more rigorous irradiation conditions are required to generate a broader range of degradation products, facilitate their structural characterization, and enable subsequent assessment of their potential toxicity using experimental or in silico approaches [8,9]. A few previous studies have investigated the forced degradation of OLO under various stress conditions. In one study, OLO was subjected to thermal stress (60 °C) and UV irradiation in the solid state, as well as acidic (0.1 M HCl), alkaline (0.1 M NaOH), neutral (water), and oxidative (3% and 10% H_2_O_2_) conditions. OLO was found to degrade under all tested conditions, and one major degradation product was proposed. However, no photodegradation products formed under UV irradiation were identified [10]. In another study, forced degradation was performed under acidic (1 M phosphoric acid), alkaline (1 M NaOH), oxidative (3% H_2_O_2_), photolytic, and thermal (80 °C) conditions. OLO exhibited degradation under thermal, oxidative, and photolytic stress, whereas only one degradation product was detected under oxidative and thermal conditions. This product was identified as OLO-related compound B according to the United States Pharmacopeia (USP) [11]. Similarly, evaluated the stability of OLO under acidic (1 M HCl), alkaline (1 M NaOH), and oxidative (30% H_2_O_2_) conditions, as well as after exposure to 200 Wh/m^2^ of UV light, 1.2 million lux·h of Vis light, 75% relative humidity at 25 °C, and elevated temperature (80 °C), oxidative degradation was the predominant degradation pathway, whereas photodegradation was limited. Two oxidative degradation products were identified and shown to correspond to USP-related compounds B and C [12]. Mahajan et al. reported approximately 10% degradation of OLO in 1 M HCl at 80 °C, 5% degradation in 1 M NaOH at 80 °C, and approximately 15% degradation following photostability testing (200 Wh/m^2^ UV light and 1.2 million lux·h Vis light). Four photodegradants were subsequently characterized by LC-TOF-MS [13]. In another study, photodegradation products of OLO were investigated in pharmaceutical formulations containing benzalkonium chloride and other excipients. The authors identified the Z- and E-isomers of carbaldehyde derivatives as photodegradation products formed in the presence of these formulation components [14]. All above identified degradants of OLO are shown in Figure 2.

**Figure 3:**
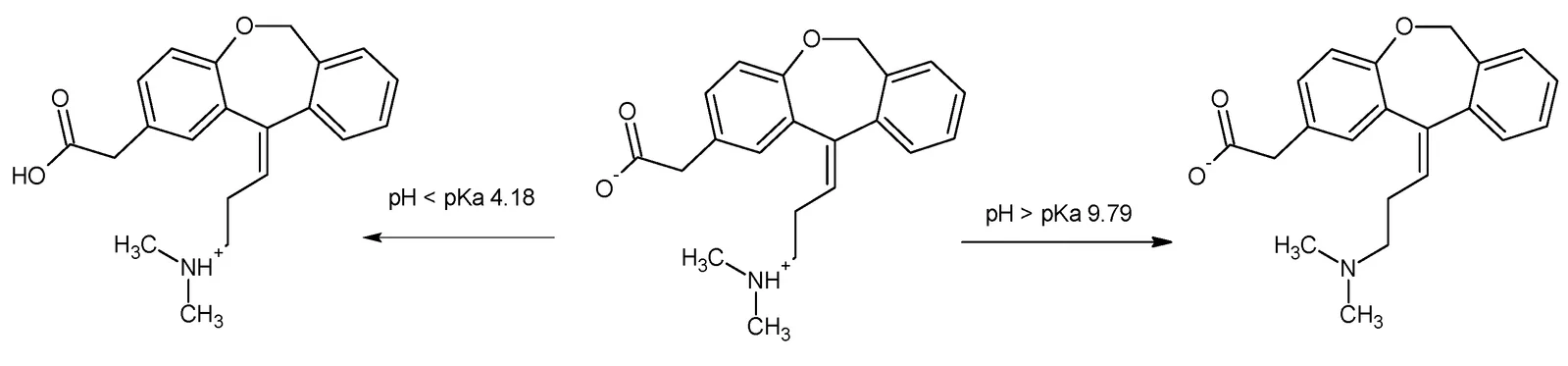
Kationic, zwitterionic and anionic forms of olopatadine (OLO).

Overall, the available literature demonstrates that OLO can be susceptible to photodegradation. However, comprehensive information on its photodegradation pathways and the structures of the resulting degradation products remains limited, particularly with respect to forced photodegradation studies performed under irradiation more stringent than those recommended by the ICH Q1B guideline (200 Wh/m^2^ of UV light and 1.2 million lux·h of visible light) [7,15]. Only a small number of photodegradation products have been structurally characterized, and their potential toxicological significance has not been systematically evaluated. A comprehensive review of the available literature also revealed a lack of information regarding the influence of pH on the photodegradation behavior of OLO. Meanwhile, the molecule possesses two ionizable functional groups, with pKa values of 4.18 for the carboxylic acid group and 9.97 for the tertiary amine group [1,2]. Consequently, OLO exists predominantly in different ionic forms depending on the pH of the medium, and its photochemical behavior may therefore vary among its cationic, zwitterionic, and anionic species (Figure 3).

The pH aspect is particularly relevant for ophthalmic formulations, which may be prepared over a relatively broad pH range using different buffering systems [16]. Therefore, the present study aimed to comprehensively investigate the photodegradation of OLO under UV/Vis irradiation across a wide pH range 1–13. Particular emphasis was placed on evaluating and comparing kinetics of photodegradation at different pH, identifying and structurally characterizing the resulting photodegradation products, and assessing their potential toxicity using two in silico approaches.

## 2. Results and Discussion

### 2.1. UV/Vis Analysis

**Figure 4:**
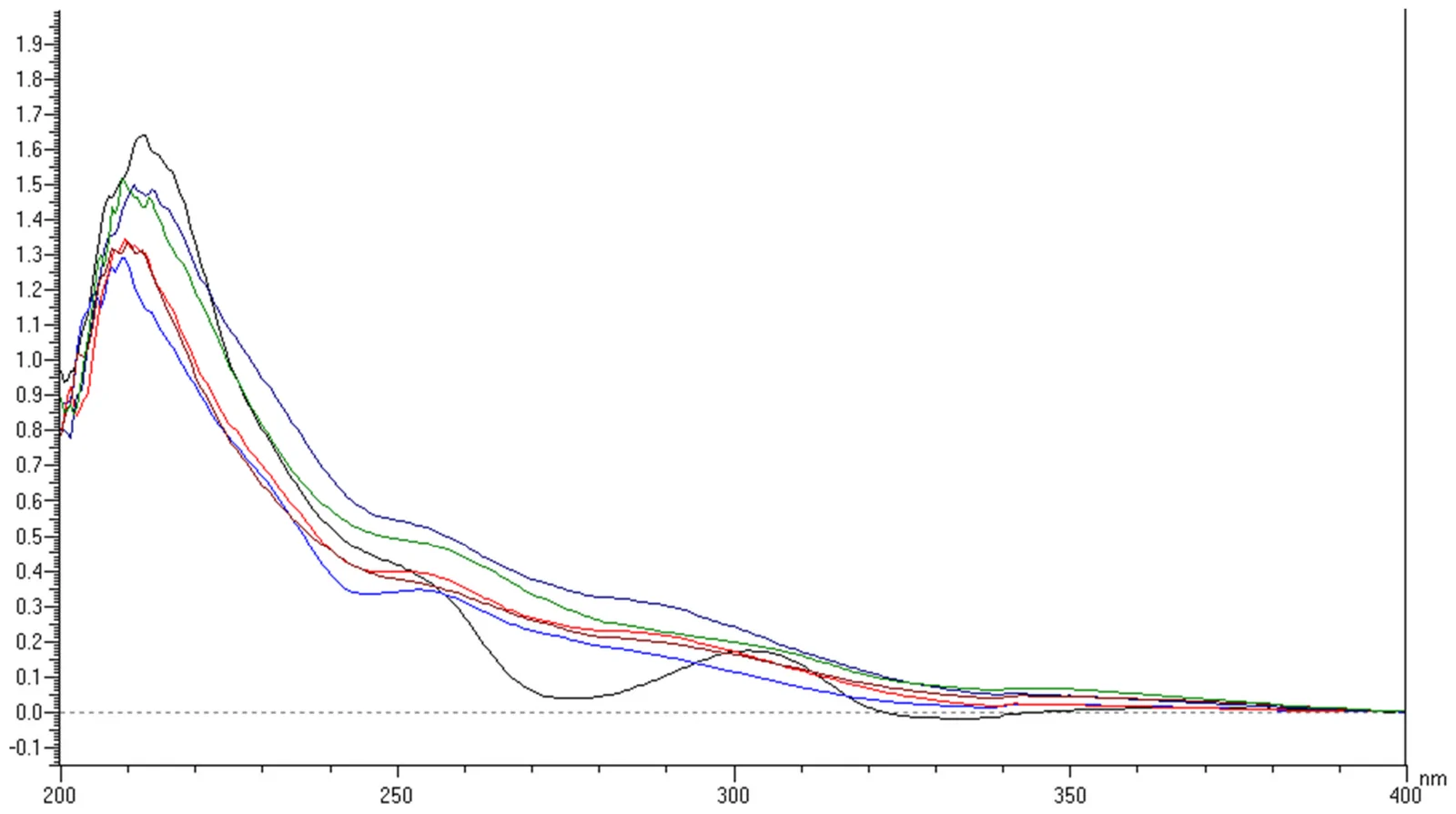
Absorption spectra of OLO samples irradiated at pH 1–13 (the spectrum of the reference solution is marked in black while the spectra of the irradiated solutions were marked as follows: pH 1 in light blue, pH 4 in red, pH 7 in dark blue, pH 10 in brown and pH in 13 green).

UV/Vis spectroscopic analysis was performed for OLO solutions over the wavelength range of 200–400 nm. The absorption spectrum of reference OLO exhibited a distinct band extending above 300 nm, with a molar extinction coefficient (MEC) exceeding 1000 M^−1^ cm^−1^, indicating that OLO is capable of absorbing near-UV radiation and may therefore be susceptible to photodegradation and phototoxicity [15]. Following UV/Vis irradiation, the absorption spectra of OLO irradiated in solutions at different pH differed markedly from those of the corresponding non-irradiated samples. Changes were observed in both the position of the absorption maxima and the shape of the absorption bands, indicating potential alterations in the molecular structure of OLO resulting from photodegradation (Figure 4).

### 2.2. Percentage Degradation and Kinetics

Quantitative determination of OLO in the irradiated samples was performed using a stability-indicating LC-UV method. It was validated for selectivity, linearity, limit of detection (LOD), limit of quantification (LOQ), precision, and accuracy, in accordance with the relevant ICH guidelines [17]. The validation results, together with the corresponding statistical assessment, are summarized in Table S1 (Section Supplementary Materials).

Kinetic calculations were based on the mean concentrations of unchanged OLO determined after each irradiation period and evaluated by linear regression of the natural logarithm of the remaining OLO concentration versus irradiation time, assuming first-order degradation kinetics. A maximum deviation of 5% from linearity was adopted as a practical acceptance criterion for the purposes of the comparative kinetic analysis. The first-order kinetic model provided a good fit to the experimental data across the investigated pH range, with *R*^2^ values of 0.9779–0.9964, with the highest observed deviation from linearity being 1.76%.

The percentage degradation of OLO and first-order kinetic parameters, including the degradation rate constant (*k*), the time required for 10% degradation (*t*_0.1_), and the half-life (*t*_0.5_) are presented in Table 1. At pH ≤ 7, degradation ranged from 45.54% (pH 7) through 62.65% (pH 4) to 62.83% (pH 1), with first-order rate constants of 3.45 × 10^−5^–5.37 × 10^−5^ s^−1^. Under alkaline conditions (pH ≥ 10), degradation increased markedly from 80.86 (pH 10) to 81.95% (pH 13), accompanied by higher rate constants of 6.14 × 10^−5^–6.91 × 10^−5^ s^−1^, respectively. Consistent with the increased degradation rates, *t*_0.5_ decreased from 0.85–0.54 h at pH ≤ 7 to 0.42–0.48 h at pH ≥ 10, while *t*_0.5_ decreased from 5.58–3.58 h to 2.79–3.14 h, respectively. Thus, our results are consistent with some extent with those reported previously for photodegradation of OLO in methanol showing *t*_0.5_ value of 3.22 h [18]. In addition, our results demonstrate that the photodegradation of OLO is pH-dependent, with alkaline and strong acidic conditions accelerating the degradation process and consequently reducing the photostability of OLO. This pronounced pH dependence can be attributed to changes in its ionization state. OLO contains two ionizable functional groups, with pKa values of 4.18 for the carboxylic acid group and 9.97 for the tertiary amine group [1,2]. As a result, the predominant molecular species changes from cationic through zwitterionic to anionic with increasing pH (Figure 3), which is likely to influence its behavior [8].

**Figure 5:**
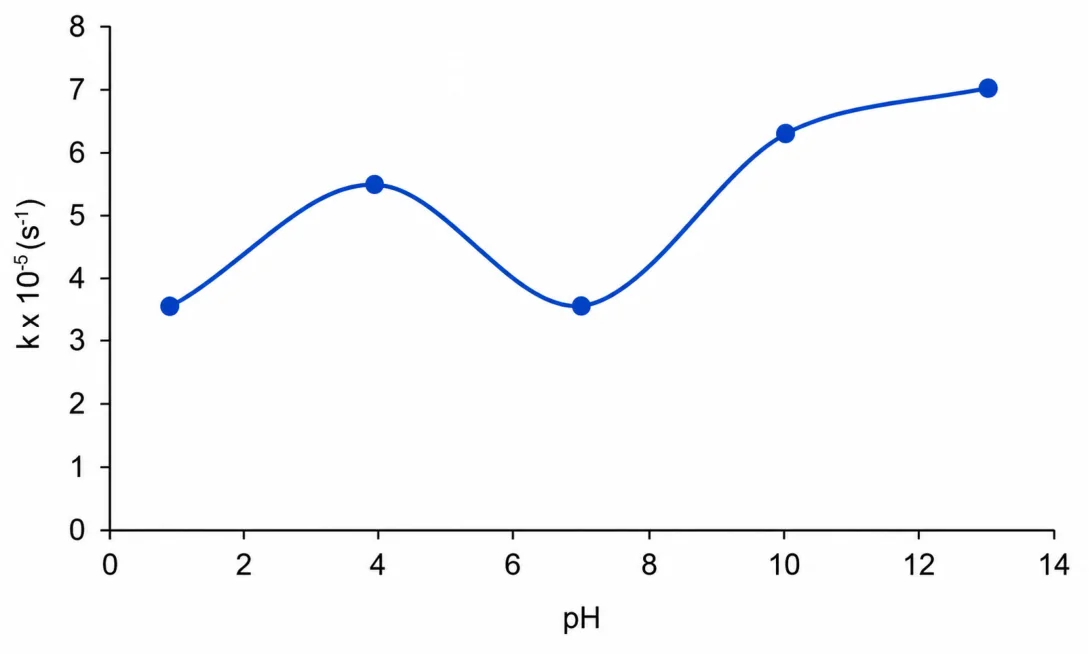
pH rate profiles for degradation of OLO under photolytic stress.

Overall, these findings confirm that light is an important factor governing degradation of OLO over the investigated pH range, with the highest degradation rates observed under acidic conditions (pH 1–4), where OLO predominantly exists in its cationic form, and, to a substantially greater extent, under alkaline conditions (pH 10–13), where its anionic form predominates (Figure 5). It was interesting to observe that final extent of OLO degradation is comparable at pH 1 and pH 4, while the calculated rate constants (*k*) differ. It indicates that degradation extent and degradation rate describe different aspects of the process. Probably, OLO undergoes photodegradation to a similar overall extent at both pH values within the investigated irradiation period, but the temporal course of degradation differs. A higher *k* indicates faster initial/overall disappearance according to the fitted kinetic model, whereas the final percentage degradation depends additionally on the duration of irradiation and possible changes in the degradation process over time [8].

In addition, photochemical reactivity of OLO was supported by our additional stability studies where samples of OLO at pH 1–13 were stored at 40 °C in the absence of light. Under these conditions, degradation at pH 1 did not exceed 40%, whereas only 0–4% degradation was observed in solutions with pH values between 4 and 13. Thus, all our findings indicate that, except under strongly acidic conditions, thermal degradation of OLO is negligible compared to photodegradation, highlighting the dominant role of light in the degradation process of zwitterionic and anionic forms of OLO. Although ophthalmic formulations are generally designed to be isohydric, with a pH close to that of the tear fluid (approximately 7.0–7.4), other variants may be used for technological reasons. Depending on the solubility and stability requirements of the particular API, ophthalmic formulations may require adjustment to a wider pH range (approximately 3.5–8.5) through the use of different buffering systems. Moreover, strong acids (e.g., HCl) or bases (e.g., NaOH) may be used during the manufacturing process to facilitate API dissolution, potentially resulting in pH conditions that can affect drug stability. Furthermore, the literature data as well as ICH Q1A(R2) guidelines recommend evaluating the stability of drug substances in ocular products under a range of conditions, including the assessment of hydrolytic and photolytic degradation across relevant pH conditions [19,20]. Therefore, the present investigation of OLO photostability over an extended pH range provides valuable information for understanding its degradation behavior and supports the development of appropriate strategies to minimize light-induced degradation. The results presented here highlight the importance of selecting suitable formulation conditions and protecting OLO-containing liquid preparations from light to maintain the product quality and therapeutic performance.

### 2.3. Identification of Olopatadine Degradation Products by UPLC-HRMS

**Figure 6:**
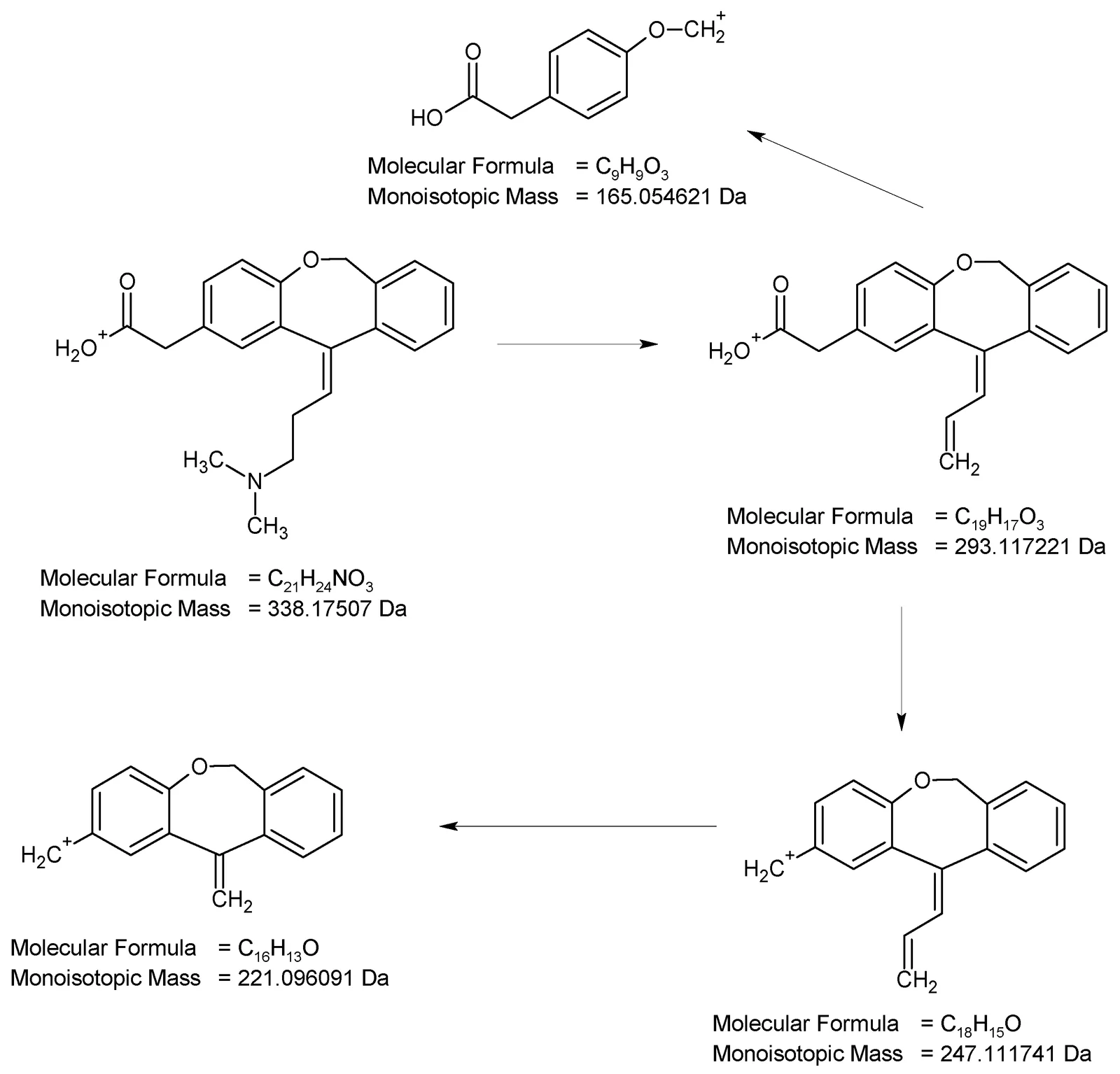
Proposed fragmentation pattern of olopatadine (OLO, Z-isomer).

The full-scan mass spectrum of the reference OLO obtained in positive ionization mode showed a protonated molecular ion at *m*/*z* 338 ([M + H]^+^). The corresponding MS/MS spectrum revealed a major product ion at *m*/*z* 293, resulting from the loss of the tertiary amine moiety. Further fragmentation generated ions at *m*/*z* 165, attributed to cleavage of the tricyclic scaffold, and *m*/*z* 247, associated with decarboxylation. The latter underwent subsequent fragmentation to produce an ion at *m*/*z* 221. These fragmentation pathways are consistent with previously reported mass spectrometric behavior of OLO [13]. The proposed fragmentation pattern of the OLO (Z-isomer) is presented in Figure 6.

**Figure 7:**
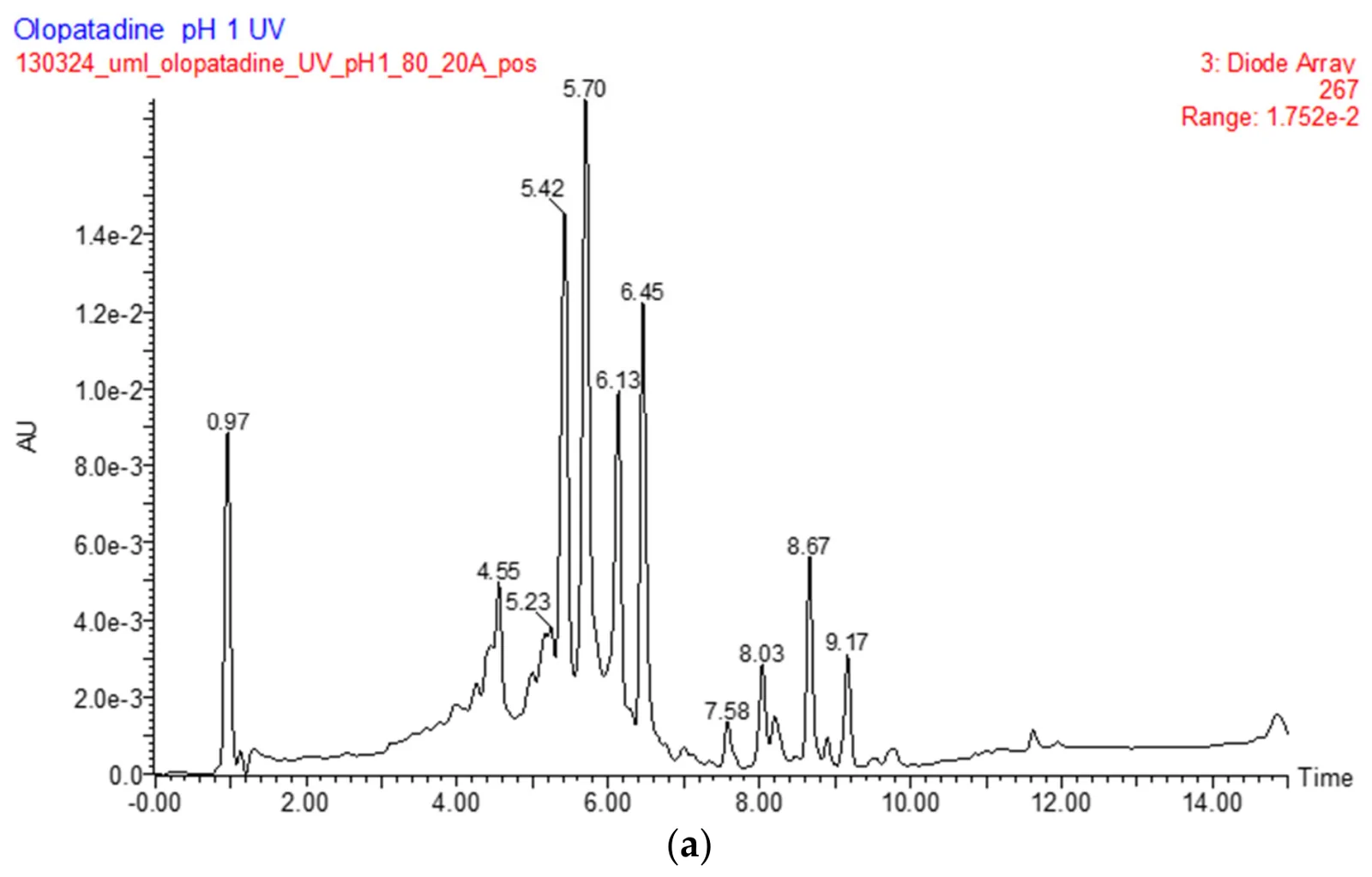

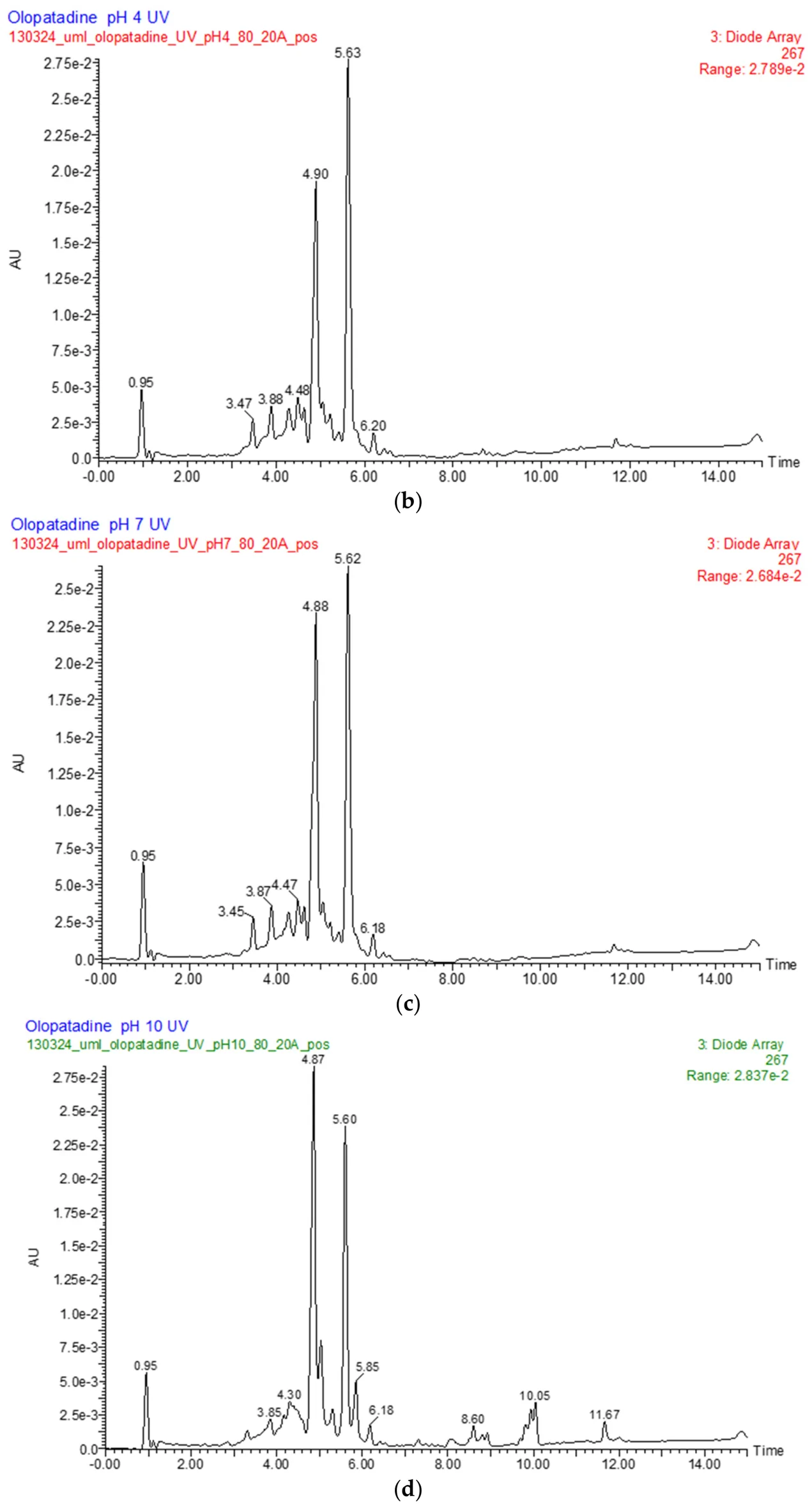

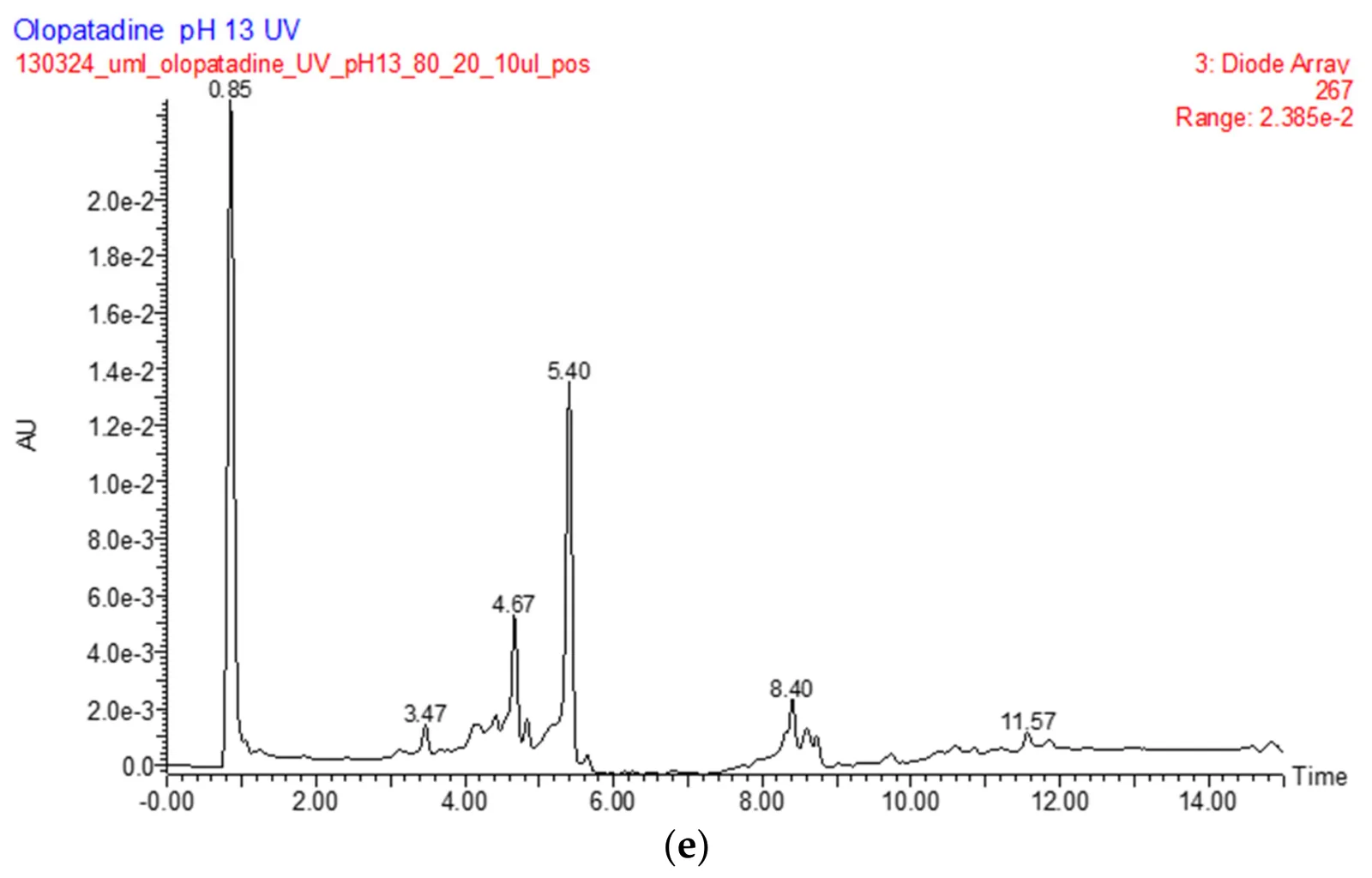
UPLC-UV chromatograms at 267 nm after irradiation of OLO: (**a**) degradation at pH 1; (**b**) degradation at pH 4; (**c**) degradation et pH 7; (**d**) degradation at pH 10 and (**e**) degradation at pH 13.

Representative UPLC-UV chromatograms of irradiated OLO solutions across the pH range 1–13 are presented in Figure 7. Analysis of the obtained HRMS data enabled the identification of twelve degradation products, and their proposed fragmentation pathways are described below.

**Figure 8:**
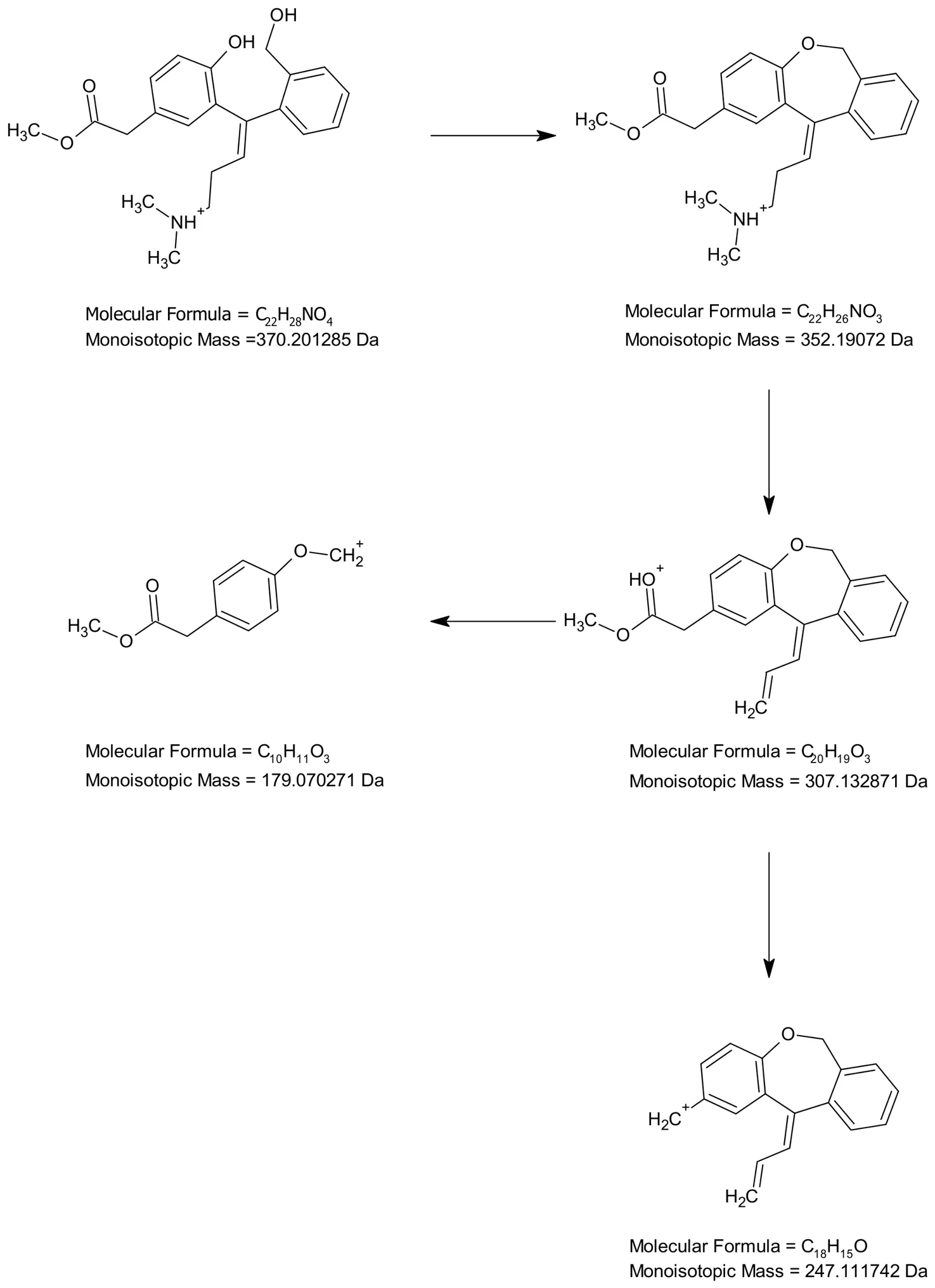
Proposed fragmentation pattern of OLO degradants 1 and 2 (OLO1 and OLO2).

For OLO samples irradiated at pH 1, 4, and 7, full-scan mass spectra of the chromatographic peaks at *t*_R_ = 4.32 and 4.45 min were acquired. The spectra revealed protonated molecular ions at *m*/*z* 370, which were assigned to the degradation products OLO1 and OLO2, respectively. These products were proposed to correspond to the E- and Z-isomers formed through opening of the oxepine ring, followed by hydroxylation leading to the formation of a 2-(hydroxymethyl)phenyl moiety. Additionally, esterification of the carboxylic acid group of OLO in the presence of methanol was proposed to occur under the applied experimental conditions. The MS/MS spectra of OLO1 and OLO2 showed product ions at *m*/*z* 352, resulting from the elimination of a protonated hydroxyl group and subsequent ring closure involving the remaining hydroxyl group. Further fragmentation of this ion generated product ions at *m*/*z* 307, attributed to the loss of the tertiary amine moiety, and *m*/*z* 247, assigned to methyl ester hydrolysis followed by decarboxylation. Moreover, both OLO1 and OLO2 spectra contained a product ion at *m*/*z* 179, which was attributed to cleavage of the C-C bond connecting the 2-(hydroxymethyl)phenyl moiety with the (3-dimethylamino)propylidene chain, resulting in the formation of a methyl 4-hydroxyphenylacetate fragment (Figure 8).

**Figure 9:**
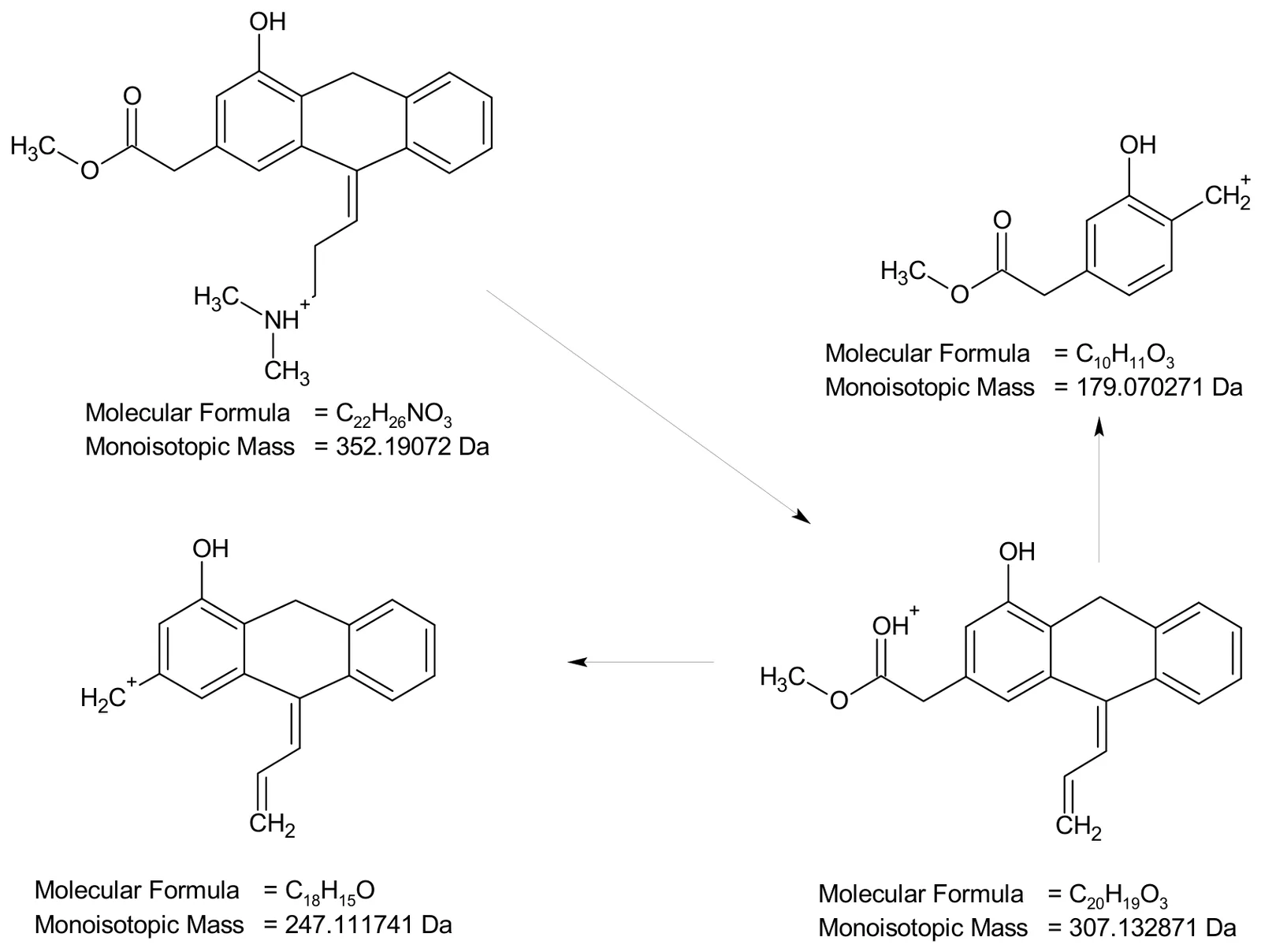
Proposed fragmentation pattern of OLO degradant 3 (OLO3).

The full-scan mass spectrum of the chromatographic peak at *t*_R_ = 5.47 min was also acquired (Figure 7), revealing a protonated molecular ion at *m*/*z* 352, which was assigned to the degradation product OLO3. This product was proposed to result from opening and subsequent rearrangement of the oxepine ring. Moreover, esterification of the carboxylic acid group of the parent OLO molecule in the presence of methanol was indicated by a CH_2_-COOCH_3_ moiety. The MS/MS spectrum of OLO3 showed product ions at *m*/*z* 307 and *m*/*z* 247, corresponding to the loss of the tertiary amine moiety and methyl ester hydrolysis followed by decarboxylation, respectively (Figure 9).

**Figure 10:**
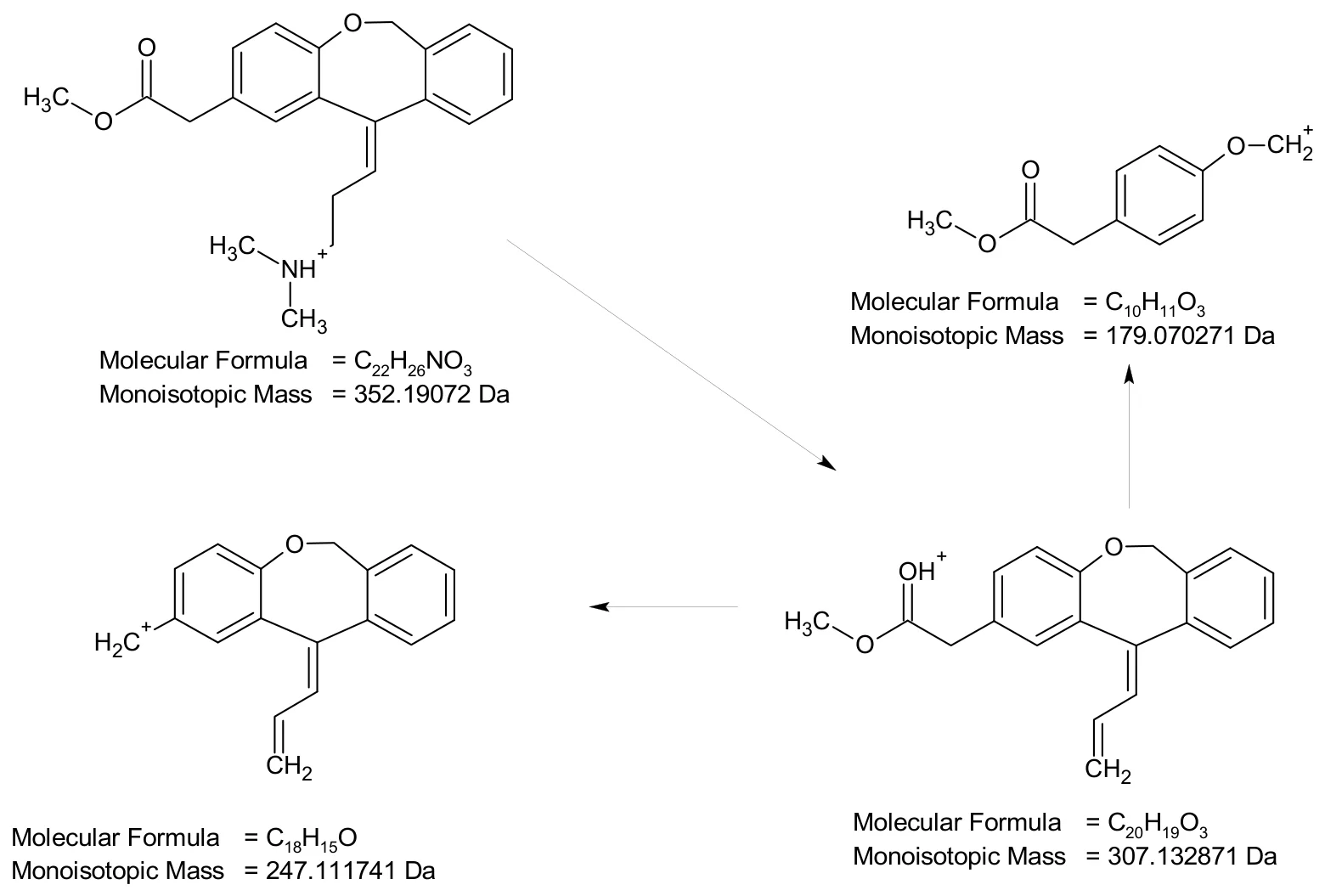
Proposed fragmentation pattern of OLO degradants 4 and 5 (OLO4 and OLO5).

The chromatographic peaks at *t*_R_ = 5.89 and 6.22 min (Figure 7) showed protonated molecular ions at *m*/*z* 352, which were assigned to the degradation products OLO4 and OLO5, respectively, in agreement with previously reported findings [13]. These compounds were proposed to correspond to the methyl ester derivatives of OLO and its E-isomer, formed through esterification of the carboxylic acid group, probably in the presence of methanol, similarly to previous results from the literature [13]. The MS/MS spectra of both isomers exhibited a product ion at *m*/*z* 307, resulting from the loss of the tertiary amine moiety. Further fragmentation generated a product ion at *m*/*z* 247, attributed to elimination of the ester group, as well as a product ion at *m*/*z* 179, which was assigned to cleavage of the tricyclic scaffold without loss of the ester moiety, leading to the formation of a methyl phenylacetate fragment (Figure 10).

**Figure 11:**
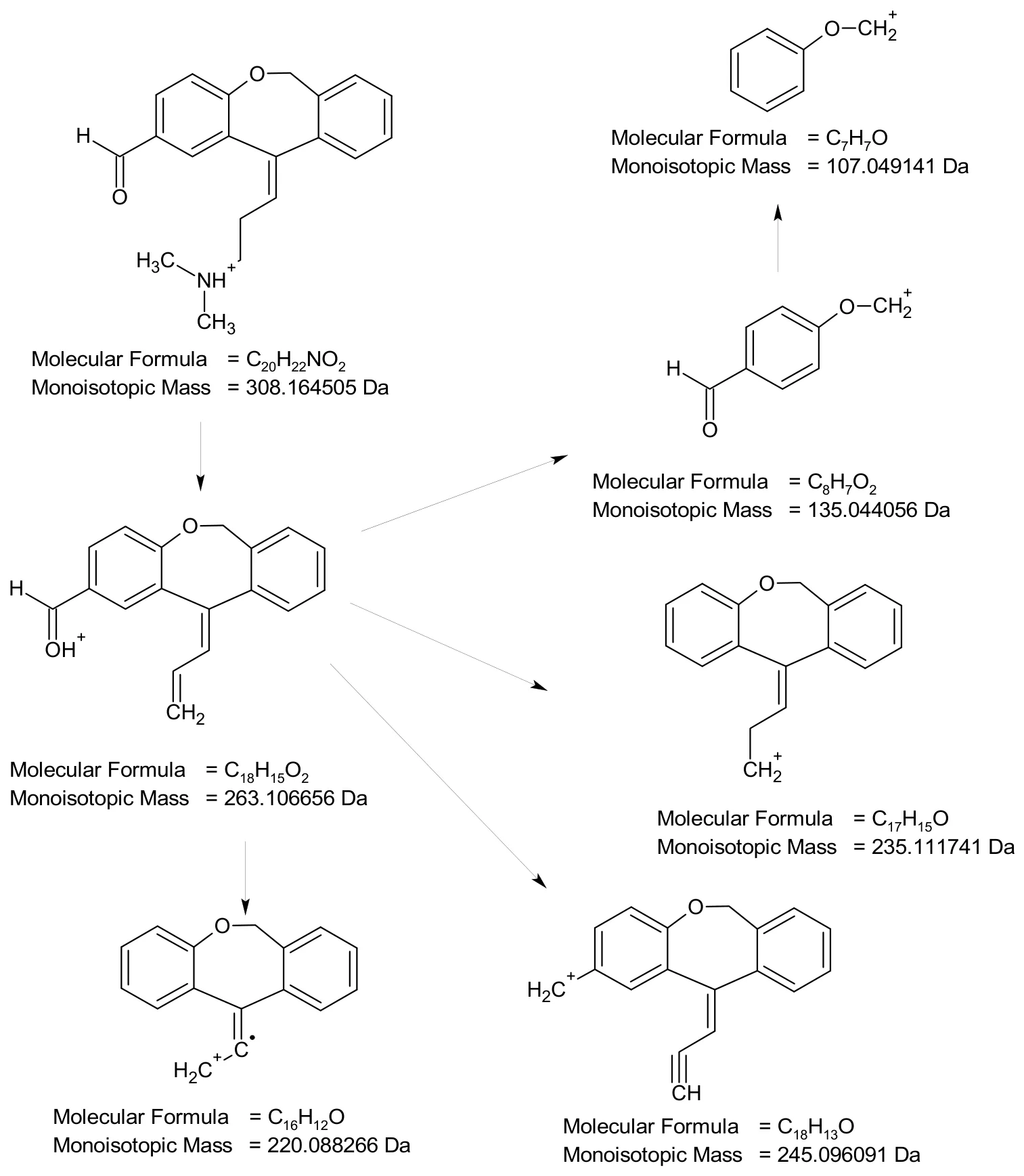
Proposed fragmentation pattern of OLO degradant 6 and 7 (OLO6 and OLO7).

Finally, full-scan mass spectra were acquired for the chromatographic peaks at *t*_R_ = 5.18 and 5.47 min (Figure 7), revealing protonated molecular ions at *m*/*z* 308. These degradation products, designated as OLO6 and OLO7, were assigned as the Z- and E-isomers of carbaldehyde derivatives of the parent OLO molecule, respectively, in agreement with previously reported findings [14]. The MS/MS spectra of both isomers exhibited a product ion at *m*/*z* 263, resulting from the loss of the tertiary amine moiety. Further fragmentation generated product ions at *m*/*z* 245, 235, and 220, which were attributed to subsequent losses involving the aldehyde group. Additionally, a product ion at *m*/*z* 135 was detected and assigned to cleavage of the tricyclic scaffold without elimination of the aldehyde moiety (Figure 11).

**Figure 12:**
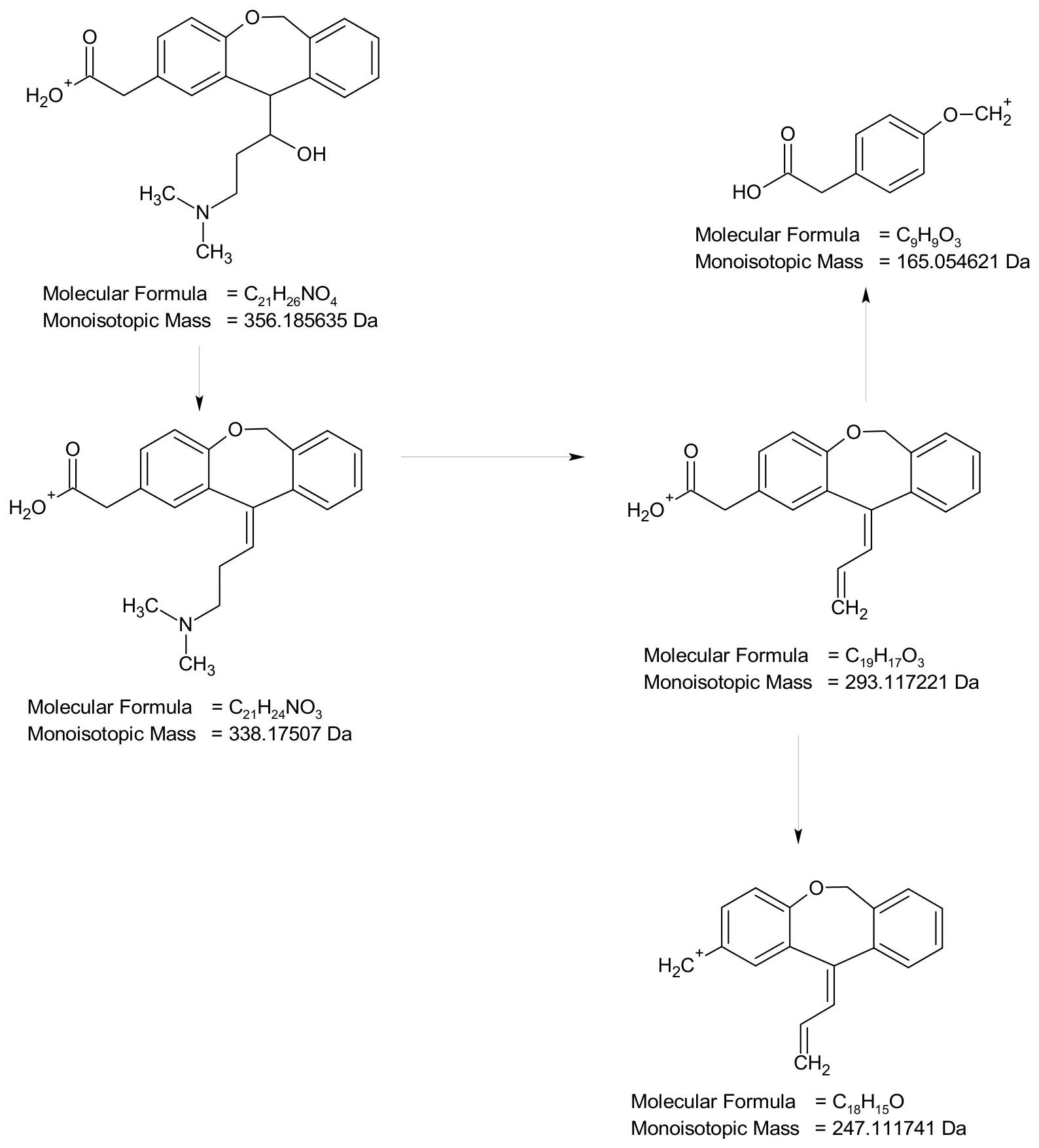
Proposed fragmentation pattern of OLO degradant 8 (OLO8).

**Figure 13:**
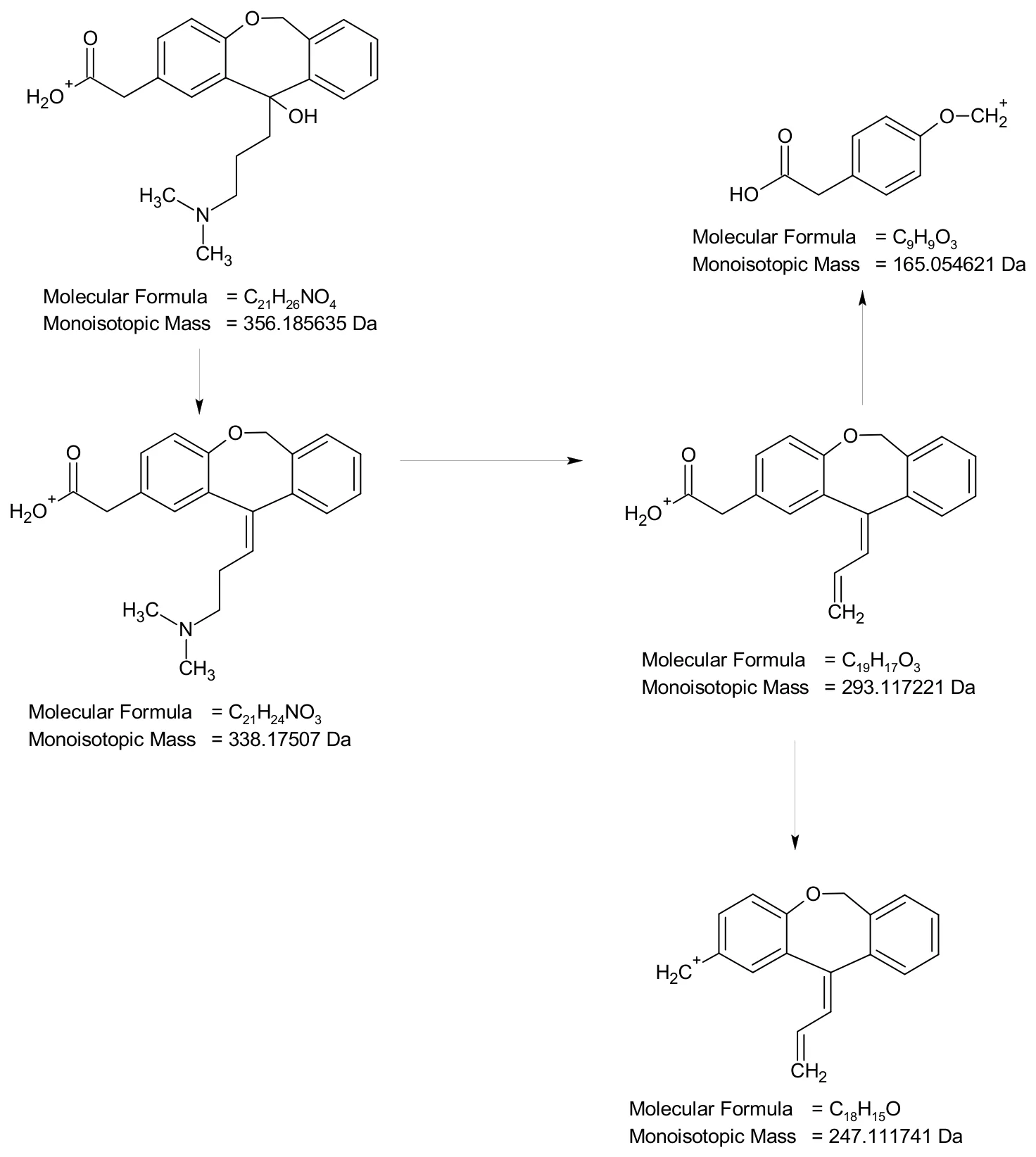
Proposed fragmentation pattern of OLO degradant 9 (OLO9).

When OLO samples irradiated at pH 4, 7, and 10 were analyzed by UPLC-HRMS/MS, chromatographic peaks at *t*_R_ = 3.31 and 4.15 min were observed (Figure 7). The corresponding full-scan mass spectra revealed protonated molecular ions at *m*/*z* 356, which were assigned to the degradation products OLO8 and OLO9, respectively. These compounds were proposed to be hydroxylation products of the parent OLO molecule, with hydroxylation occurring either within the propylidene chain or the oxepine ring. The MS/MS spectra of OLO8 and OLO9 exhibited product ions at *m*/*z* 338, resulting from the loss of the hydroxyl group. Further fragmentation of this ion generated product ions at *m*/*z* 293, attributed to the elimination of the tertiary amine moiety, and *m*/*z* 165, assigned to cleavage of the tricyclic scaffold accompanied by the release of a phenylacetic acid fragment. Additionally, a product ion at *m*/*z* 247 was detected and attributed to decarboxylation (Figure 12 and Figure 13).

**Figure 14:**
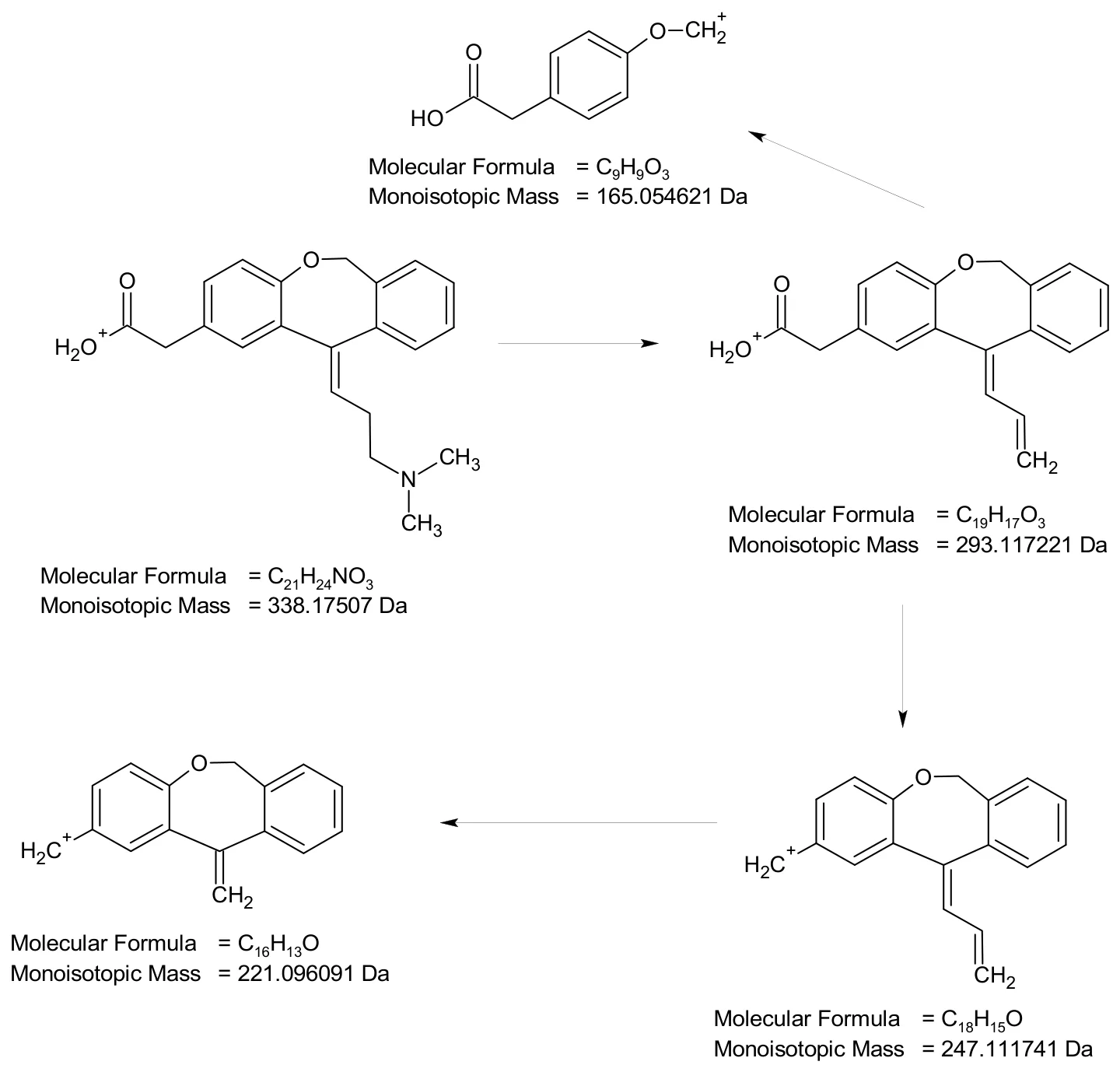
Proposed fragmentation pattern of OLO degradant 10 (OLO10).

Analysis of the HRMS data obtained for OLO samples irradiated at pH 4, 7, 10, and 13 enabled the identification of an additional degradation product, designated as OLO10, in agreement with previously reported findings [13]. This compound was assigned as the E-isomer of OLO and was detected at *t*_R_ = 5.38 min (Figure 7). The MS/MS spectrum of OLO10 exhibited a fragment ion at *m*/*z* 293, corresponding to the loss of the tertiary amine moiety. Subsequent fragmentation yielded ions at *m*/*z* 165, attributed to the (4-methoxyphenyl)acetic acid fragment formed by cleavage of the tricyclic scaffold, and *m*/*z* 247, assigned to decarboxylation followed by generation of the fragment ion at *m*/*z* 221. These fragmentation pathways were consistent with those observed for the parent OLO (Z-isomer). The proposed fragmentation pathway of OLO10 is presented in Figure 14.

**Figure 15:**
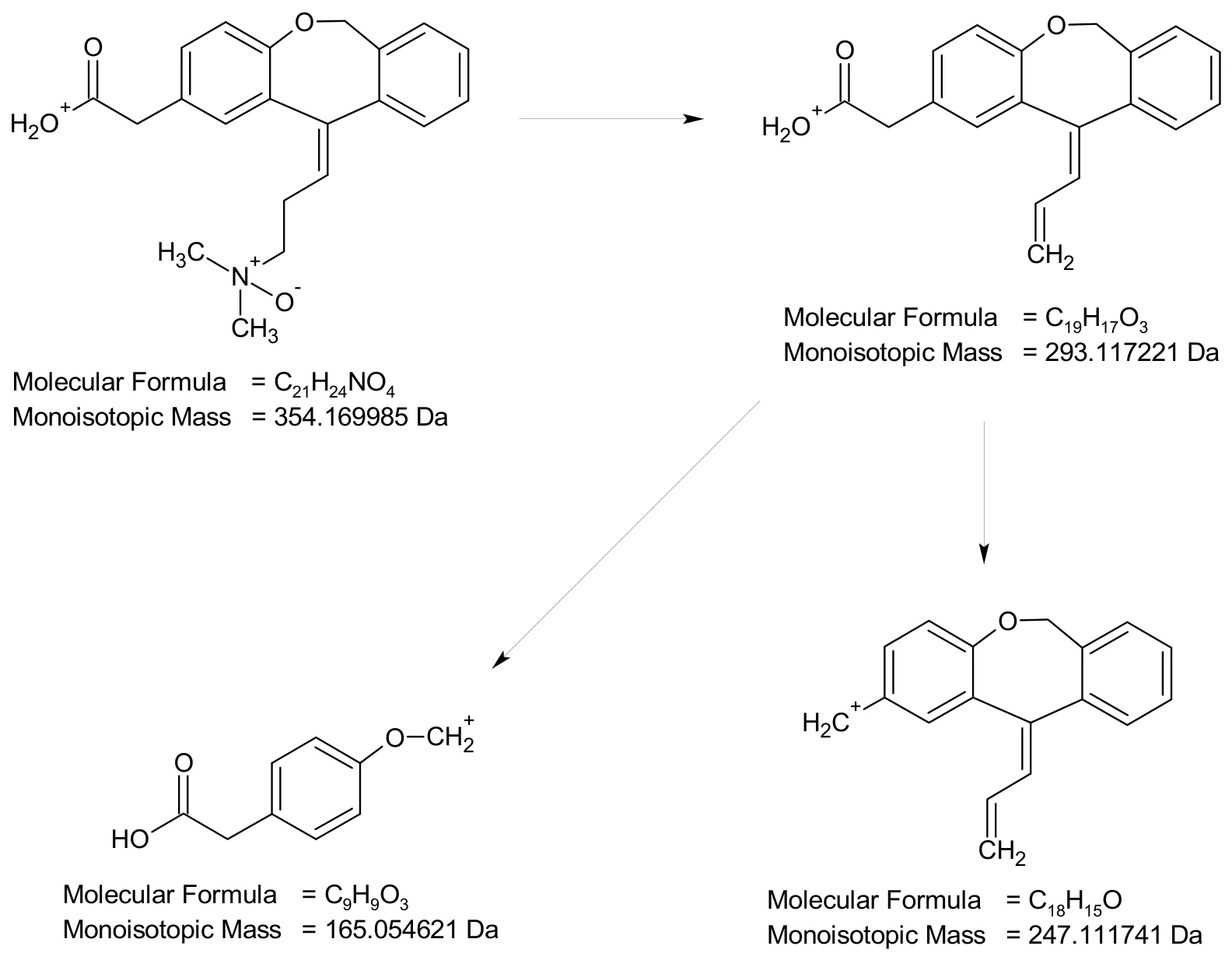
Proposed fragmentation pattern of OLO degradant 11 and 12 (OLO11 and OLO12).

Analysis of the HRMS data obtained for OLO samples degraded at pH 10 and 13 enabled the identification of two additional degradation products, designated as OLO11 and OLO12 in agreement with previously reported findings from forced oxidative conditions [11,12]. These compounds corresponded to chromatographic peaks at *t*_R_ = 4.86 and 5.67 min, respectively (Figure 7), and exhibited protonated molecular ions at *m*/*z* 354. OLO11 and OLO12 were proposed to result from oxidation of the tertiary amine nitrogen atom in OLO and its E-isomer, respectively. The MS/MS spectra of both compounds showed a fragment ion at *m*/*z* 293, generated by the elimination of the oxidized tertiary amine moiety. Subsequent fragmentation proceeded through several pathways, including decarboxylation leading to the formation of the ion at *m*/*z* 247, decarboxylation followed by cyclization resulting in the ion at *m*/*z* 232, and cleavage of the tricyclic scaffold with release of the (4-methoxyphenyl)acetic acid fragment corresponding to the ion at *m*/*z* 165 (Figure 15).

### 2.4. Identification of Olopatadine Degradation Products by NMR

**Figure 16:**
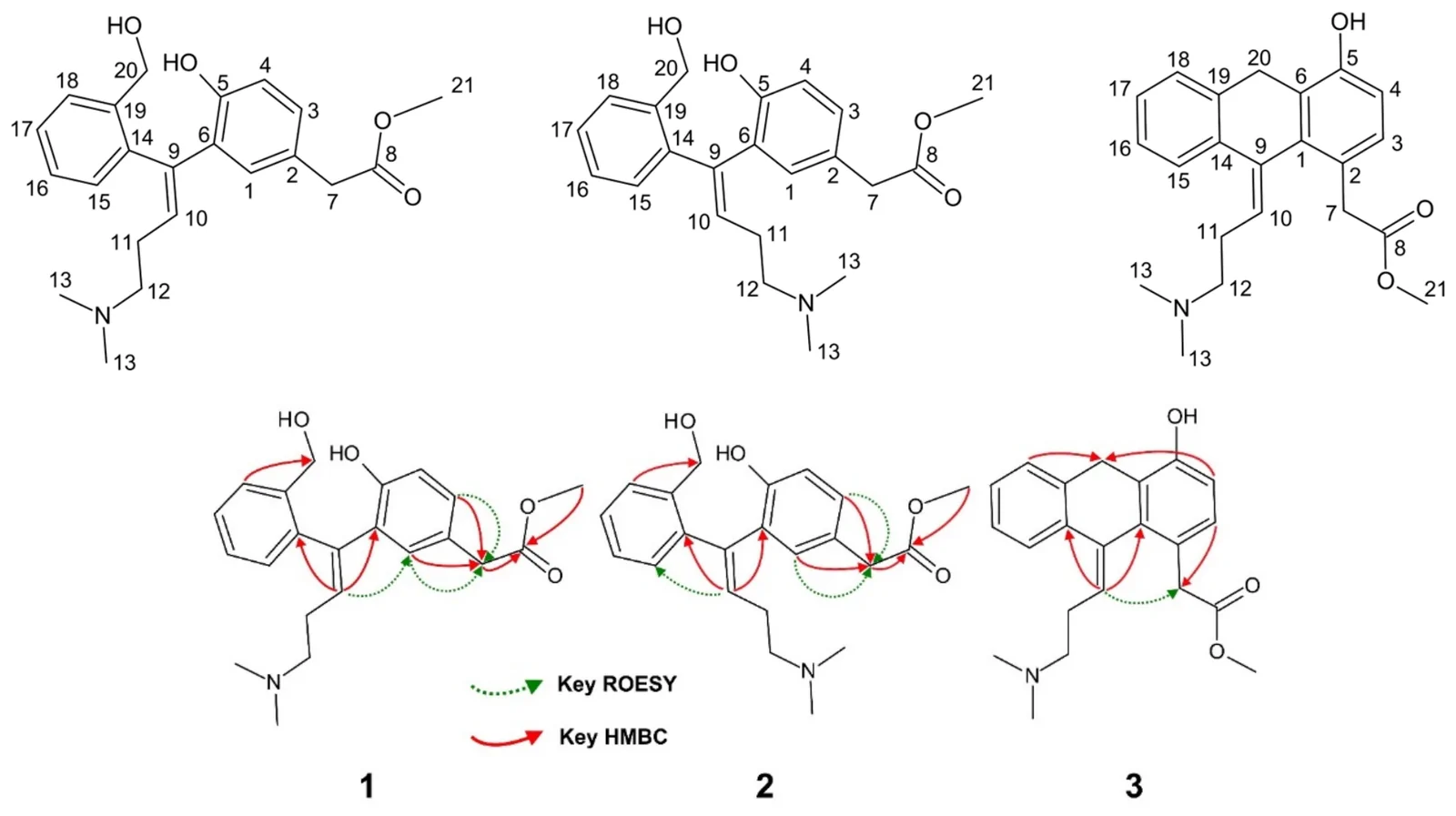
The structures of OLO1, OLO2 and OLO3 and their key HMBC and ROESY correlations.

Preparative separation was performed on OLO samples irradiated in an aqueous solution at pH 1, resulting in the isolation of six fractions. Structural analysis of these fractions by NMR spectroscopy enabled the identification and confirmation of six photodegradation products: OLO1, OLO2, OLO3, OLO4, OLO5, and OLO6. The confirmed structures of three newly described OLO1, OLO2, and OLO3, together with the most significant long-range ^1^H–^13^C correlations from HMBC experiments and through-space ^1^H–^1^H correlations from 1D ROESY spectra, are presented in Figure 16. The ^1^H and ^13^C NMR data for OLO1–OLO3, their derivatives, and the parent OLO molecule are summarized in Table 2. Additionally, the corresponding 1D and 2D NMR spectra are provided in the Section Supplementary Materials.

Analysis of the 1D and 2D NMR spectra of OLO1, together with a comparison of its NMR data with those of OLO, allows the identification of this compound. As shown in Table 2, the NMR data of OLO1 exhibit many features similar to those of the parent OLO. Briefly, the downfield region of the ^1^H NMR spectrum (see 6.0–8.0 ppm region in Figure S1) contains signals that can be attributed to two aromatic rings and an alkene moiety. Two sets of proton signals, namely δ_H_ 7.08 (1H, dd, *J* = 7.4, 1.3 Hz, H-15), 7.24 (1H, td, *J* = 7.5, 1.2 Hz, H-16), 7.30 (1H, td, *J* = 7.5, 1.5 Hz, H-17) and 7.51 (1H, dd, *J* = 7.6, 0.6 Hz, H-18), and δ_H_ 6.74 (1H, d, *J* = 8.3 Hz, H-4), 6.91 (1H, dd, *J* = 8.3, 2.2 Hz, H-3) and 6.71 (1H, d, *J* = 2.3 Hz, H-1), originate from a disubstituted aromatic ring and a trisubstituted aromatic ring, respectively. The alkene moiety, giving rise to a single proton signal at δ_H_ 6.07 (1H, t, *J* = 7.4 Hz, H-10), is part of the 4-dimethylaminobut-1-enyl fragment. The proton resonances of the remaining groups within this fragment are observed at δ_H_ 2.04 (2H, m, H-11), 2.46 (2H, t, *J* = 6.9 Hz, H-12), and 2.19 (6H, s, H-13), and are unequivocally assigned to two distinct methylene groups and two equivalent methyl groups. However, in contrast to the parent OLO, the alkene moiety of OLO1 adopts an *E* configuration. This stereochemical assignment is based on the through-space correlation occurring between the olefinic proton H-10 and the aromatic proton H-1, as observed in the 1D selective ROESY spectrum obtained by selective irradiation of H-10 (Figure S5A). Further analysis of the NMR spectra of OLO1 allows the identification of its remaining characteristic structural features. Unlike in OLO, its aromatic rings are linked exclusively through the olefinic carbon C-9. This connectivity can be deduced from the HMBC correlations of the alkene proton H-10 to the aromatic ^13^C nuclei giving rise to resonances at δ_C_ 137.9 (C-14) and 128.7 (C-6) (see Figure S4). The second linkage between the aromatic rings, i.e., the Ar-CH_2_-O-Ar fragment present in OLO, is replaced in OLO1 by CH_2_OH and OH groups attached to different aromatic rings. Although signals corresponding to the two OH groups are not observed in the ^1^H NMR spectrum, possibly due to the rapid chemical exchange of the OH protons [21,22], the changes in the chemical shifts in the CH_2_ group are consistent with the proposed structural modification. As can be seen, both the ^1^H and ^13^C signals of this methylene group, observed at δ_H_ 4.37 (1H, brs, H-20) and 4.28 (1H, brs, H-20), and at δ_C_ 60.8 (C-20), are shifted upfield relative to the corresponding resonances of OLO (see the NMR data for OLO in Table 2). This variation is consistent with the replacement of the -O-Ar moiety by an -OH group, as the former typically causes greater deshielding of the adjacent CH_2_ group [23]. Finally, the distinct proton signal at δ_H_ 3.55 (3H, s, H-21) can be attributed to the CH_3_ group of the CH_2_COOCH_3_ moiety attached to one of the aromatic rings.

Comparison of NMR spectra and NMR-related data of OLO1 and OLO2 (see columns 1 and 2 in Table 2) clearly demonstrates that they are isomers differing solely in the configuration of the alkene bond. OLO2 has the *Z* configuration, as evidenced by the through-space correlation between its alkene proton giving rise to signal at δ_H_ 5.67 (1H, t, *J* = 7.2 Hz, H-10) and adjacent aromatic proton with signal occurring at δ_H_ 7.05 (1H, dd, *J* = 7.6, 1.3 Hz, H-15) (see 1D selective ROESY spectra of proton H-15 in Figure S11A).

Analysis of OLO3 ^1^H spectrum shows that, unlike in case of the parent OLO as well as OLO1 and OLO2, signals originating from only 6 aromatic protons are present in its downfield region (see 5.5–8.0 ppm region in Figure S13). ^1^H resonances occurring at δ_H_ 7.37 (1H, m, H-15), 7.27 (1H, td, *J* = 7.4, 1.2 Hz, H-16), 7.23 (1H, td, *J* = 7.4, 1.3 Hz, H-17) and 7.37 (1H, m, H-18) arise from disubstituted aromatic ring. Two doublets corresponding to mutually coupled protons at δ_H_ 6.70 (1H, d, *J* = 8.3 Hz, H-4) and 6.97 (1H, d, *J* = 8.3 Hz, H-3) originate from second aromatic ring. The absence of additional aromatic signals indicates that the latter ring is tetra-substituted. As the J-coupling constant of H-4 and H-3 is equal to 8.3 Hz, it can be concluded that those nuclei are placed in ortho positions to each other. Moreover, the chemical shift in H-4 indicates that it is located at the ortho position relative to an oxygen-containing electron-donating group. The ortho substituent with respect to proton H-3 is CH_2_-COOCH_3_ moiety. Such connectivity can be deduced from HMBC correlation occurring between H-3 and C7. Additional long range ^1^H-^13^C correlations occurring between H-4/C-20, H-18/C-20, H-10/C-1 and H-10/C-14 indicate that both rings are connected via alkene and methylene moieties (see HMBC spectra in Figures S16 and S17). Finally, through space-correlation between olefinic proton H-10 and H-7 from CH_2_ in CH_2_-COOCH_3_ fragment indicate *Z* geometry of this alkene bond (see selective ROESY spectrum in Figure S18A).

Finally, methyl ester of OLO (OLO4) and its E-isomer (OLO5) as well as E-isomer of OLO (OLO10) were isolated and identified. Their NMR data (see Table S2 in Section Supplementary Materials) is consistent with the literature reports [3].

### 2.5. Photodegradation Products of Olopatadine

Overall, twelve OLO photodegradation products were proposed (Table 3), seven of which, i.e., OLO1, OLO2, OLO3, OLO8, OLO9, OLO11 and OLO12, are reported here for the first time. Although previous studies have demonstrated the susceptibility of OLO to photodegradation, its photochemical behavior has remained only partially characterized. Under ICH photostability conditions (200 Wh/m^2^ of UV light and 1.2 million lux·h of visible light) [7], four degradation products were detected following irradiation of OLO in methanolic solution, corresponding to our OLO4, OLO5, OLO10, and OLO11 [13]. In another investigation, the Z- and E-isomers of OLO carbaldehyde derivatives were reported [14], corresponding to OLO6 and OLO7 identified herein (Figure 2). Thus, the present findings are consistent with previous reports regarding the formation of OLO4, OLO5, OLO6, OLO7, OLO10, and OLO11. Oxidation of the tertiary amine nitrogen atom of OLO has previously been reported under oxidative stress conditions, yielding OLO USP related compound B [11,12], which corresponds to OLO11 identified in the present study. However, we demonstrate for the first time that this oxidation product, together with its E-isomer (OLO12), can also be generated under UV/Vis irradiation. Importantly, the present work substantially expands the current understanding of OLO photodegradation by identifying seven previously unreported photoproducts, OLO1, OLO2, OLO3, OLO8, OLO9, OLO11 and OLO12, three of which, OLO1, OLO2 and OLO3, are confirmed by our both HRMS and NMR analyses. The identified photodegradation products were formed through pathways involving oxepine ring opening and rearrangements, hydroxylation, and esterification. Another important aspect revealed by this study is the propensity of OLO to undergo geometric isomerization during photodegradation. Consequently, careful monitoring and control of OLO-related impurities, particularly geometric isomers with unknown pharmacological and toxicological properties, are essential to ensure the quality, safety, and efficacy of OLO-containing pharmaceutical products [24]. In the case of OLO ophthalmic and nasal formulations, such information may be particularly valuable for the optimization of manufacturing and sterilization processes [12].

### 2.6. Risk of Toxicity Using in Silico Methods

In silico toxicity assessment of the identified degradation products of OLO was performed using the OSIRIS Property Explorer and Toxtree approaches available online [25,26], revealing several potentially relevant structural toxicity alerts. The OSIRIS methodology predicts toxicity risks based on the presence of specific structural fragments within a molecule. These structural alerts are identified by comparison with compounds previously associated with specific toxicity endpoints, including mutagenicity, tumorigenicity, irritation, and reproductive toxicity [27]. Analysis of the structures of OLO1, OLO2, OLO3, OLO4, and OLO5 revealed the presence of a prop-1-ene-1,1-diyldibenzene fragment associated with a high risk of reproductive toxicity. OLO6 and OLO7 were additionally predicted to exhibit a high risk of irritation due to the presence of a benzaldehyde moiety, while also showing a reproductive toxicity alert. Furthermore, OLO9 was identified as a compound with a medium tumorigenicity risk according to the OSIRIS prediction model. In addition, the Toxtree analysis classified all above-mentioned OLO degradation products as well as OLO8, as the compounds with a Class III level of toxicity concern. According to this classification, such compounds lack structural features supporting an initial presumption of safety and may contain reactive functional groups associated with potential toxic effects. This prediction is based on the structure-based Threshold of Toxicological Concern (TTC) approach, which categorizes chemicals according to their structural characteristics and corresponding exposure thresholds [28,29,30]. We would like to emphasize that the in silico toxicity predictions presented herein should be regarded as preliminary hazard-screening information rather than evidence of actual toxicological risk. What is more, the formation of specific degradation products under forced-degradation conditions, although indicative of the potential degradation pathways of the respective API under extreme stress conditions, does not imply that these compounds will occur at comparable levels, or necessarily form at all, under normal manufacturing, storage, or use conditions. Accordingly, the structures identified in the present study should primarily be regarded as a qualitative library of potential OLO degradation products. This library may support subsequent targeted quantitative monitoring of relevant degradants during formal stability studies and facilitate the prioritization of compounds for further toxicological assessment. Although topical administration of H1-receptor antagonists such as OLO generally results in limited systemic exposure and minimal systemic effects, potential local toxicity associated with their degradation products should still be carefully considered. This is particularly relevant for photodegradation products that may form during storage or use and could contribute to local adverse effects, including irritation. Moreover, regulatory guidelines for topical medicinal products emphasize the importance of evaluating toxicity at the site of administration, especially for formulations applied to small and sensitive areas such as the eye or nasal mucosa. Therefore, these guidelines recommend comprehensive assessment of degradation product toxicity for topical formulations, similar to the requirements applied to systemically administered drugs [31].

## 3. Materials and Methods

### 3.1. Materials

Olopatadine hydrochloride (OLO), methanol, acetonitrile, dimethyl sulfoxide (DMSO), deuterated dimethyl sulfoxide (DMSO-d_6_), dichloromethane, formic acid, and chromatography-grade water were purchased from Merck (Darmstadt, Germany) or J.T. Baker (Center Valley, PA, USA). Boric acid, hydrochloric acid and phosphoric acid, potassium and sodium hydroxide, potassium dihydrogen phosphate, and sodium salts, including sodium chloride, disodium hydrogen phosphate, and sodium dihydrogen phosphate, were obtained from POCh (Gliwice, Poland). Water used for the preparation of buffer solutions was purified using a Milli-Q water purification system (Merck, Darmstadt, Germany). Phosphate buffer of pH 4 (0.5 M; potassium dihydrogen phosphate/phosphoric acid), phosphate buffer of pH 7 (0.063 M; disodium hydrogen phosphate/sodium dihydrogen phosphate/phosphoric acid), and borate buffer of pH 10 (0.4 M; boric acid/sodium hydroxide) were prepared in accordance with the Polish Pharmacopoeia XII. The ionic strength was adjusted when necessary to a constant value 0.5 M using a 4 M NaCl solution. These buffers were used to prepare samples for forced degradation study performed under UV/Vis irradiation.

### 3.2. UV/Vis and LC-UV Quantitative Methods

Spectrophotometric measurements were performed using a CE6600 UV/Vis double-beam spectrophotometer (Cecil Instruments Ltd., Milton, UK). Quantitative LC-UV analysis was carried out using a chromatographic system (Waters UK Sales, Elstree, England) equipped with a model 515 pump, a Rheodyne 20 µL injector, and a model 2487 diode array detector (DAD), controlled by Empower 3 software (Waters). Chromatographic separation was achieved using a LiChrospher^®^ RP-8 end-capped column (125 × 4.0 mm, 5 µm; Merck) with an isocratic mobile phase consisting of methanol, acetonitrile, and phosphoric acid (50:50, *v*/*v*, with 0.2% phosphoric acid) at a flow rate of 1.0 mL/min. Detection was performed at 267 nm. The selectivity of the method was evaluated by determining OLO in the presence of its photodegradation products. Calibration curves were prepared using working solutions obtained from an OLO stock solution (1 mg/mL). Calibration equations (*y* = *ax* + *b*) were calculated by plotting peak areas against the corresponding OLO concentrations. The standard deviations of the slope (*a*) and intercept (*b*) were used for calculation of the limits of detection (LOD) and quantification (LOQ). Accuracy and precision were assessed at low, medium, and high OLO concentration levels.

### 3.3. Photodegradation of Olopatadine in Solutions

Volumes of 1 mL of OLO stock solution (2 mg/mL) were mixed with 1 mL of the respective solvent, i.e., 0.1 M HCl (pH 1), buffer solutions at pH 4, 7, and 10, or 0.1 M NaOH (pH 13), in quartz glass-stoppered dishes. The prepared samples were exposed to UV/Vis irradiation for 0.25, 0.5, 1.0, 2.5, and 10 h, corresponding to irradiation doses of 592, 1080, 2163, 10,800, and 21,599 kJ/m^2^, with their corresponding control samples, which were prepared and handled under identical experimental conditions but protected from light by aluminum foil. Irradiation was performed using a Suntest CPS Plus chamber (Atlas, Linsengericht, Germany) equipped with a temperature control unit maintained at 35 ± 2 °C. Following the degradation experiments, the samples were neutralized, when necessary, using 0.1 M NaOH (pH 1), 0.1 M HCl (pH 13), 1 M NaOH (pH 4) and 1 M HCl (pH 10). Finally, All samples were diluted to a final volume of 10 mL with methanol, and analyzed by UV/Vis spectrophotometry, LC-UV and UPLC-HRMS/MS analysis.

For spectrophotometric evaluation the samples were diluted 20-fold with methanol, and UV spectra were recorded over the wavelength range of 200–400 nm using the appropriate blank solutions. The resulting spectra were then compared with those of the corresponding control samples prepared under identical conditions but protected from light. For quantitative determination by LC-UV, the samples were diluted two-fold with methanol and injected onto the chromatographic column in triplicate for each pH condition and each irradiation time point. The peak areas were recorded for both the light-exposed samples and the corresponding control samples prepared under identical conditions but protected from light, allowing the concentration of OLO remaining unchanged after light exposure to be determined. Subsequent kinetic calculations were based on the corresponding mean concentrations of unchanged OLO determined after each irradiation period. Kinetic parameters were evaluated by linear regression of the natural logarithm of the remaining OLO concentration versus irradiation time, assuming first-order degradation kinetics. A maximum deviation of 5% from linearity was adopted as a practical acceptance criterion for the comparative kinetic analysis. The resulting linear regression equations were used to calculate the photodegradation rate constant (*k*) and the times corresponding to 10% and 50% degradation of OLO (*t*_0.1_ and *t*_0.5_, respectively). The percentage degradation of OLO was calculated from the concentration of unchanged substance relative to the corresponding control sample.

### 3.4. UPLC-HRMS/MS for Identification of Degradation Products

UPLC-MS analysis was performed using an Acquity UPLC I-Class chromatograph coupled with a Synapt G2-S HDMS mass spectrometer (Waters Inc., Milford, MA, USA), equipped with an electrospray ionization (ESI) source and a quadrupole time-of-flight (qTOF) mass analyzer. Chromatographic separation was achieved using an Acquity UPLC BEH C18 column (2.1 × 100 mm, 1.7 µm; Waters). Gradient elution was performed at a flow rate of 0.3 mL/min. The mobile phase composition was initially set at 20% eluent B and increased linearly to 80% B over 10 min, followed by an isocratic step at 80% B for 3.5 min and subsequent re-equilibration to the initial conditions. Eluent A consisted of 0.1% formic acid in water, while eluent B consisted of methanol and acetonitrile containing formic acid (50:50, *v*/*v*, with 0.2% formic acid). UV chromatograms were recorded at 267 nm. High-resolution mass spectra were acquired in positive ion mode using the following parameters: capillary voltage, 2.5 kV; desolvation gas flow, 600 L/h; desolvation temperature, 350 °C; sampling cone voltage, 20 V; source offset voltage, 20 V; and source temperature, 120 °C. Data were collected in full-scan mode over an *m*/*z* range of 100–1200. Leucine-enkephalin solution was used as the lock-spray reference compound for mass accuracy correction. The instrument was controlled, and data acquisition and processing were performed using MassLynx V4.2 software (Waters).

### 3.5. Preparative Isolation of Degradation Products

Volumes of 1 mL of OLO stock solution (200 mg/mL) prepared in a methanol/DMSO (1:1, *v*/*v*) were mixed with 1 mL of 0.1 M HCl (pH 1) in quartz glass-stoppered dishes. The samples were exposed to UV/Vis irradiation for 35 h using the solar simulator described above, corresponding to an irradiation dose of 70,197 kJ/m^2^. After irradiation, the samples were neutralized with 0.1 M NaOH. Subsequently, liquid–liquid extraction was performed to remove inorganic components from the degraded samples prior to chromatographic separation. The irradiated solution containing OLO degradation products was diluted with 20 mL of water and extracted three times with 5 mL portions of dichloromethane. The combined organic phases were collected and evaporated to dryness using a CentriVap Benchtop Vacuum Concentrator (Labconco, Kansas City, MO, USA). The resulting residue was dissolved in 5 mL of a DMSO/water mixture (1:1, *v*/*v*), and the obtained solution was subjected to preparative chromatographic separation. Preparative separation was performed using an LC-UV system equipped with an SPD-20AV UV/Vis detector and an FRC-10A automatic fraction collector (Shimadzu, Kyoto, Japan), fitted with a Luna C18 column (150 × 10 mm, 5 µm; Phenomenex, Torrance, CA, USA). Chromatographic separation was achieved under isocratic conditions using a mobile phase consisting of 20% solvent B and 80% solvent A. Solvent A was water containing 1% formic acid, while solvent B was acetonitrile containing 1% formic acid. The total run time was 27 min, with a mobile phase flow rate of 4.0 mL/min. UV detection was performed at 220 nm. The collected fractions were subsequently evaporated under reduced pressure to remove the mobile phase.

### 3.6. NMR Spectra

NMR spectra were recorded using an ASCEND 600 MHz spectrometer equipped with a BBO probe (Bruker, Mannheim, Germany). The isolated fractions were dissolved in 0.6 mL of DMSO-d_6_. One-dimensional ^1^H and ^13^C NMR spectra, as well as two-dimensional ^1^H–^1^H COSY, ^1^H–^13^C HSQC, ^1^H–^13^C HMBC, and one-dimensional ROESY spectra, were acquired using standard Bruker pulse sequences and acquisition parameters. The residual proton signal of DMSO-d_6_ was used for chemical shift calibration.

### 3.7. Methodology for Toxicity Assessment

The potential toxicological hazards of OLO and its degradation products were evaluated independently using two computer programs available online, i.e., OSIRIS Property Explorer and Toxtree (version 3.1.0) [25,26]. For each compound, the chemical structure proposed on the basis of LC-HRMS/MS and/or NMR analyses was converted into an appropriate two-dimensional molecular representation and SMILES notation. Each structure was entered individually into the molecular structure editor and verified prior to analysis. In OSIRIS Property Explorer, toxicity predictions were generated automatically after submission of the verified molecular structure. Four toxicity-related endpoints provided by the software, namely mutagenic, tumorigenic, irritant, and reproductive toxicity risks, were recorded for each compound. The results were interpreted according to the qualitative, color-coded risk categories generated by the software. Properties associated with a high risk of undesirable effects were indicated in red, whereas green indicated a low predicted risk and drug-conforming behavior [27]. For the Toxtree analysis, the Cramer rules decision tree was selected to classify the compounds into structural toxicity classes (Cramer Classes I, II, and III) on the basis of their structural characteristics and the corresponding anticipated level of toxicological concern. Class I comprises structures considered to have a low level of toxicological concern, Class II represents an intermediate level of concern, and Class III includes structures for which a higher level of toxicological concern is anticipated [28].

### 3.8. Statistical Methods

All statistical analyses were performed using Statistica 13 software (TIBCO Software Inc., Palo Alto, CA, USA).

## 4. Conclusions

The present study provides a comprehensive characterization of the photodegradation behavior of OLO under UV/Vis irradiation. Quantitative LC-UV analyses were performed to investigate photodegradation kinetics under different pH conditions, while the resulting degradation products were characterized by UPLC-HRMS/MS, isolated by preparative chromatography where feasible, structurally confirmed by NMR spectroscopy, and assessed for their potential toxicity using in silico methods. The integration of these complementary analytical and computational approaches provides a robust framework for investigating drug photostability and evaluating the potential safety implications of photodegradation products. The investigation of OLO photostability over a broad pH range (1–13) provides valuable insight into its pH-dependent degradation behavior and may support the development of appropriate strategies to minimize light-induced degradation in OLO-containing liquid formulations. However, because the experiments were conducted under deliberately severe stress conditions, the resulting degradation profiles were used primarily for comparative assessment of OLO photodegradation at different pH values and for the qualitative identification and structural characterization of degradation products. Accordingly, the relative abundance of individual degradation products observed under these forced degradation conditions should not be considered representative of their expected levels under formal stability testing or normal storage conditions. Among the twelve OLO degradation products detected in this study, seven structures were proposed for the first time, namely OLO1, OLO2, OLO3, OLO8, OLO9, OLO11 and OLO12. Of these seven newly described degradation products, OLO1, OLO2, and OLO3 were initially assigned on the basis of HRMS data and subsequently confirmed following their isolation and structural characterization by NMR spectroscopy. Owing to the limited amounts of material available, the remaining newly identified degradation products, i.e., OLO8, OLO9, OLO11 and OLO12, could not be independently confirmed by NMR spectroscopy and were therefore assigned solely on the basis of HRMS data. Consequently, these structural assignments should be regarded as tentative and require further confirmation using complementary analytical techniques. Nevertheless, reporting these tentatively identified degradation products and including them in a preliminary in silico toxicity assessment was considered valuable, as this approach provides an initial indication of their potential toxicological relevance and may help prioritize degradation products for further structural characterization and experimental toxicological evaluation. The in silico toxicity assessment performed using OSIRIS Property Explorer and Toxtree approaches indicated that several degradation products contained structural features associated with alerts for potential reproductive toxicity, irritation, or tumorigenicity. However, these predictions should be interpreted as preliminary hazard alerts rather than as definitive evidence of toxicity. Moreover, the toxicological assessment was based on two relatively simple structure-based in silico prediction methods and therefore has inherent limitations. Additional computational approaches, together with appropriate in vitro and, where justified, in vivo studies, would be required to establish the toxicological profiles and biological relevance of the identified photodegradation products. Despite these limitations, the present findings substantially expand the current knowledge of OLO photostability and provide valuable information relevant to the characterization and control of OLO photodegradation products in ophthalmic and nasal formulations. The results also provide a foundation for further investigation of the toxicological significance of OLO photodegradation products and may support the development of appropriate formulation, manufacturing, packaging, storage, and quality-control strategies for OLO-containing pharmaceutical products.

## Figures and Tables

**Table 1:** Photodegradation of olopatadine (OLO) in solutions of pH 1–13.

pH	Degradation [%]	y = ax + b	*R* ^2^	*k* [s^−1^]	*t*_0.1_ [h]	*t*_0.5_ [h]
1	62.83	y = −0.0009x + 4.1445	0.9818	3.45 × 10^−5^	0.85	5.58
4	62.65	y = −0.0014x + 4.4663	0.9779	5.37 × 10^−5^	0.54	3.58
7	45.54	y = −0.0009x + 4.5129	0.9924	3.45 × 10^−5^	0.85	5.58
10	80.86	y = −0.0016x + 3.8918	0.9964	6.14 × 10^−5^	0.48	3.14
13	81.95	y = −0.0018x + 3.9668	0.9893	6.91 × 10^−5^	0.42	2.79

**Table 2:** NMR data (DMSO-d_6_, ^1^H 600 MHz and ^13^C 150 MHz) for OLO1, OLO2, OLO3 and OLO.

	OLO1		OLO2		OLO3		OLO	
No.	^1^H	^13^C	^1^H	^13^C	^1^H	^13^C	^1^H	^13^C
1	6.71, d (2.3)	131.0	6.83, d (2.3)	131.8	-	140.6	7.07, m ^c^	132.5
2	-	124.7	-	124.7	-	123.1	-	127.9
3	6.91, dd (8.3, 2.2)	129.1	6.99, dd (8.3, 2.3)	129.6	6.97, d (8.3)	130.3	7.06, m ^c^	130.7
4	6.74, d (8.3)	116.4	6.77, d (8.3)	116.3	6.70, d (8.3)	113.3	6.76, d (8.0)	119.5
5	-	154.0	-	153.7	9.54, brs, (-OH)	152.8	-	154.1
6	-	128.7	-	127.2	-	120.8	-	123.4
7	3.43, s	39.8	3.52, s	39.7	3.71, d (16.7)	38.6	3.47, s	40.6
					3.89, d (16.7)			
8	-	172.4	-	172.4	-	173.0	-	173.6
9	-	137.9	-	137.6	-	135.0	-	139.5
10	6.07, t (7.4)	130.4	5.67, t (7.2)	131.3	5.72, t (6.7)	128.8	5.66, t (7.1)	130.7
11	2.04, m	27.4	2.17, m	27.8	2.38, m ^a^	27.4	2.56, m	27.3
					2.59, m			
12	2.46, t (6.9)	58.4	2.44, t (7.3)	58.6	2.42, m ^a^	59.1	2.59, m	58.4
13	2.19, s	44.8	2.16, s	45.0	2.16, s	45.1	2.26, s	44.7
14	-	137.9	-	142.0	-	136.9	-	145.4
15	7.08, dd (7.4, 1.3)	129.9	7.05, dd (7.6, 1.3)	129.4	7.37, m ^b^	128.7	7.26, d (7.5)	126.3
16	7.24, td (7.5, 1.2)	126.5	7.15, td (7.6, 1.4)	126.5	7.27, td (7.4, 1.2)	125.9	7.36, m ^d^	129.6
17	7.30, td (7.5, 1.2)	127.2	7.21, td (7.5, 1.3)	126.8	7.23, td (7.4, 1.6)	127.1	7.32, td (7.3, 1.2)	128.0
18	7.51, dd (7.6, 0.6)	126.5	7.43, dd (7.6, 0.6)	127.4	7.38, m ^b^	127.2	7.36, m ^d^	128.2
19	-	140.6	-	140.1	-	137.9	-	133.9
20	4.28, brs	60.8	4.49, s	61.2	3.29, d (17.6)	29.3	5.11, brs	69.8
	4.37, brs				4.14, d (17.6)			
21	3.55, s	52.0	3.57, s	52.0	3.61, s	52.1	-	-

^a^, ^b^, ^c^, ^d^ Overlapping multiplet.

**Table 3:** Photodegradation products of OLO based on HRMS and NMR analyses.

No.	Structures		Molecular Mass	Irradiation at pH
OLO1 OLO2 (E and Z-isomers)	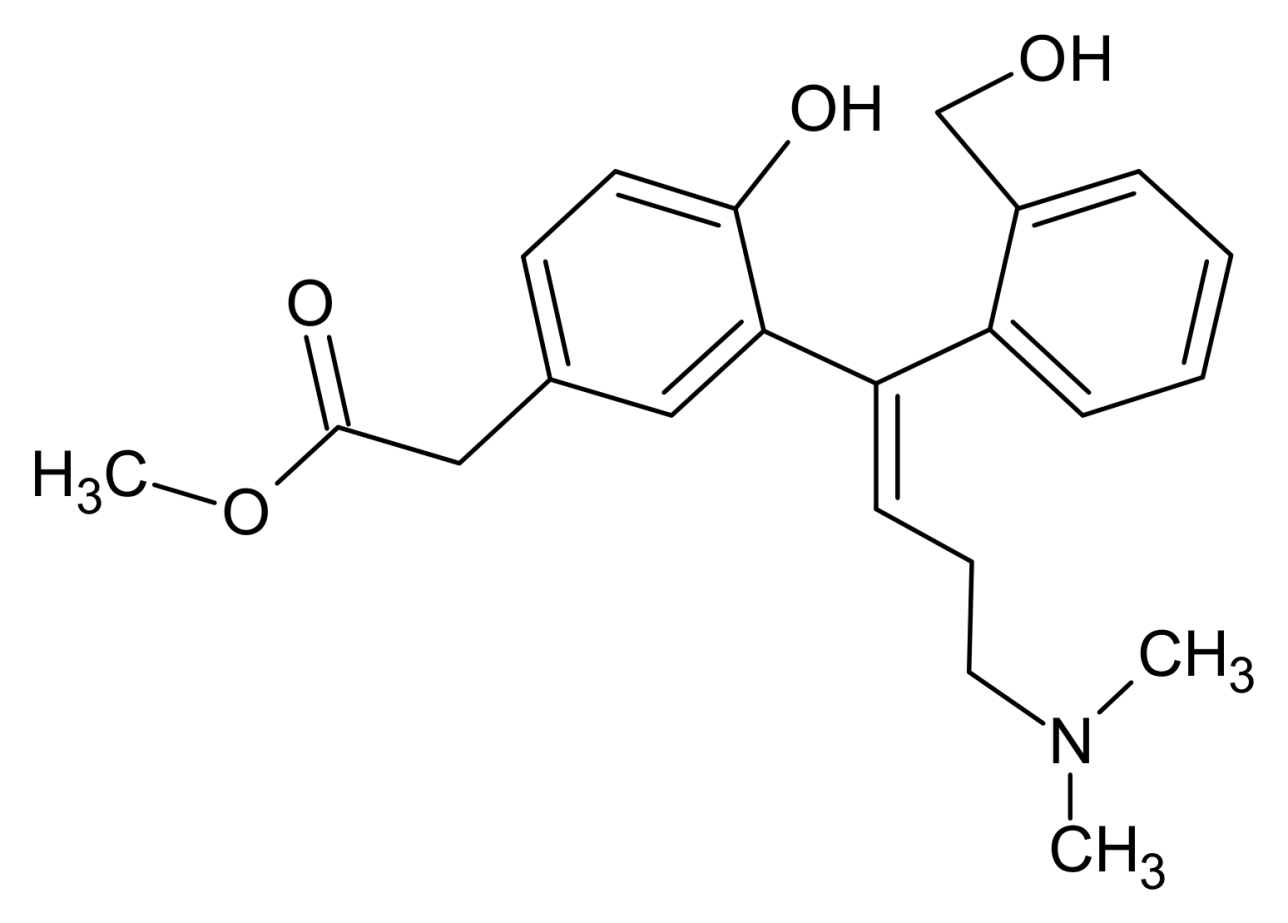	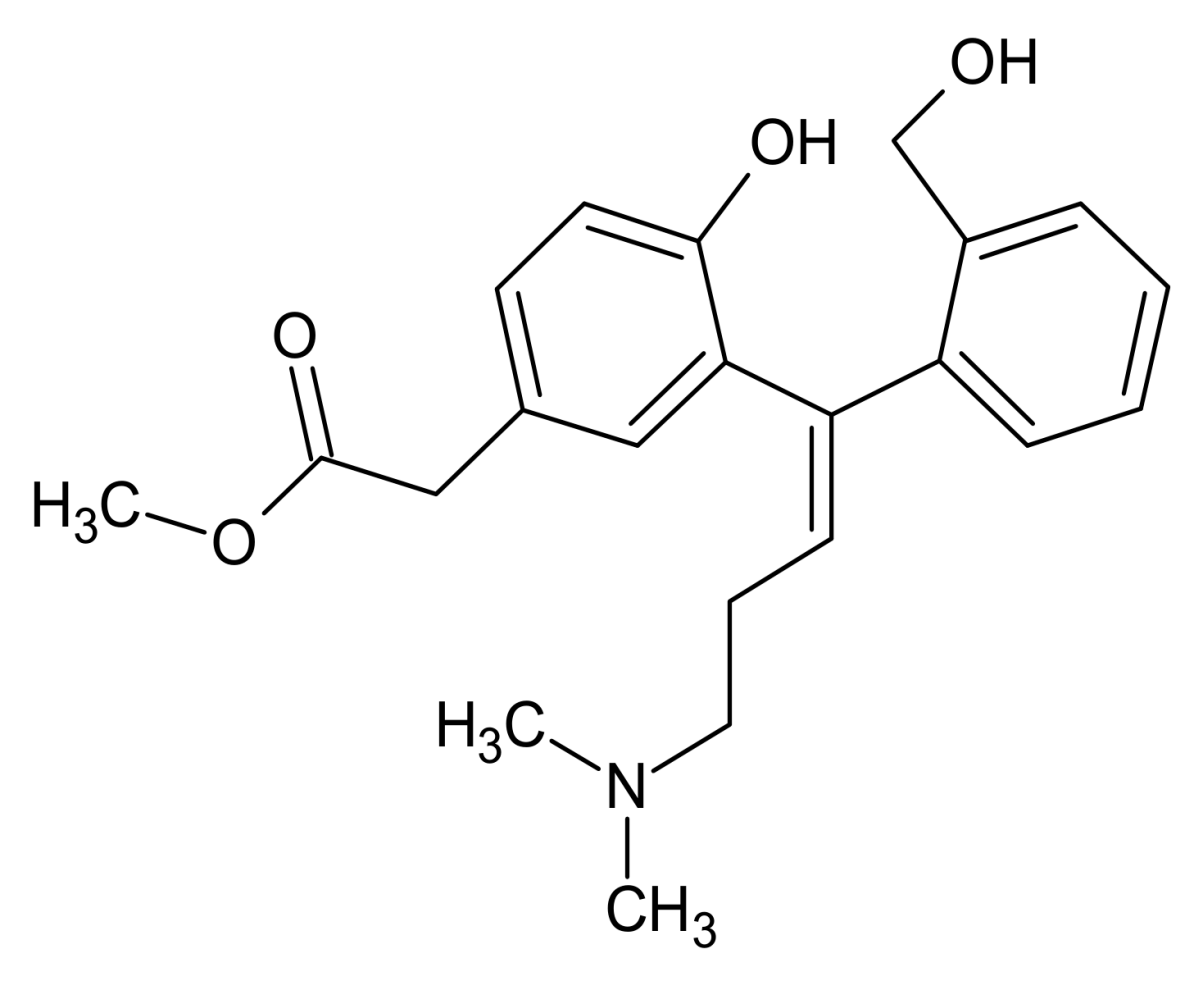	369	1, 4, 7
OLO3 (Z-isomer)	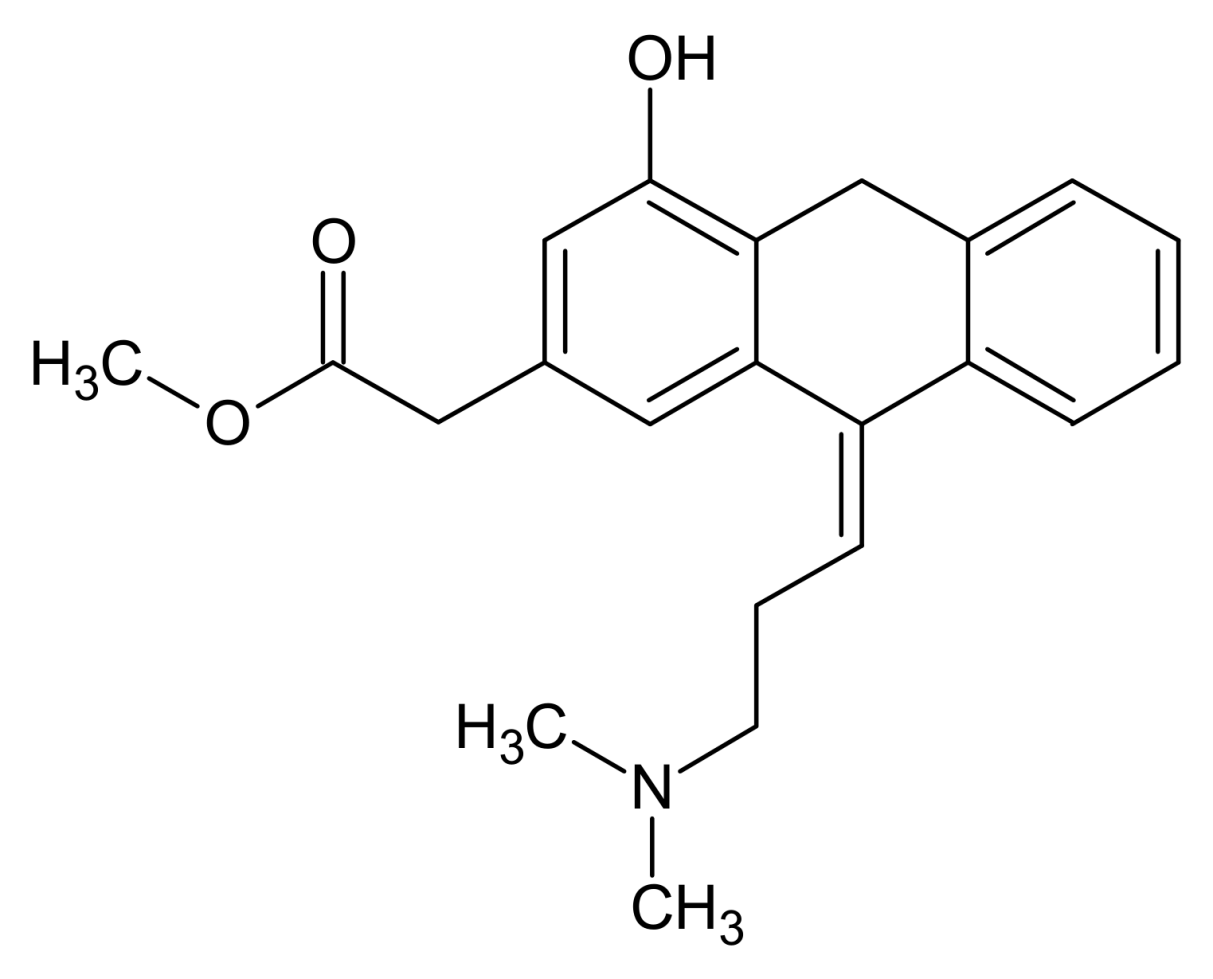		351	1, 4, 7
OLO4 OLO5 (Z and E-isomers)	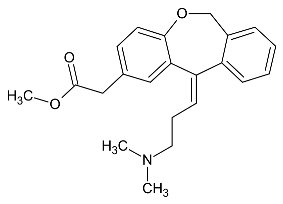	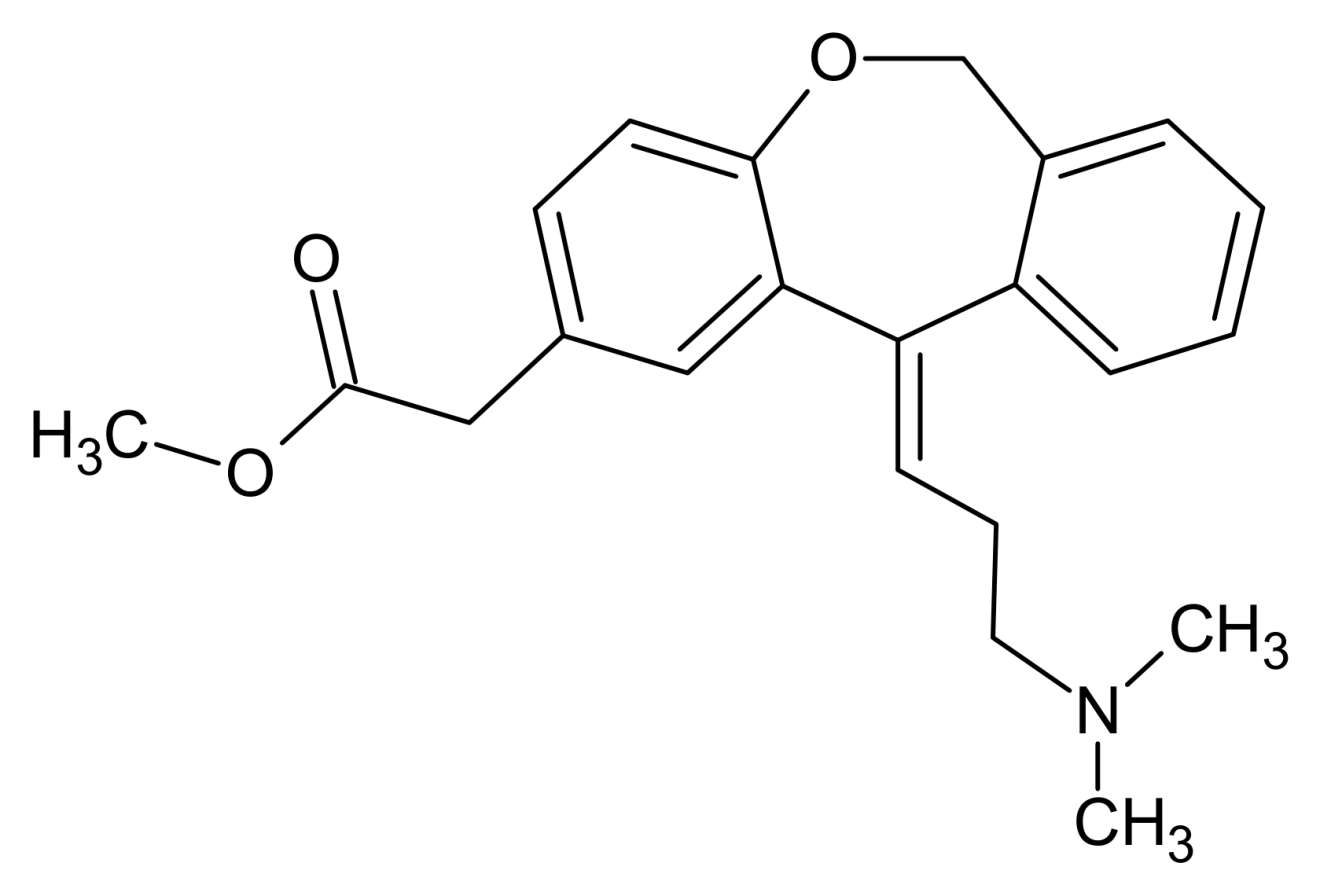	351	1, 4, 7
OLO6 OLO7 (Z and E-isomer)	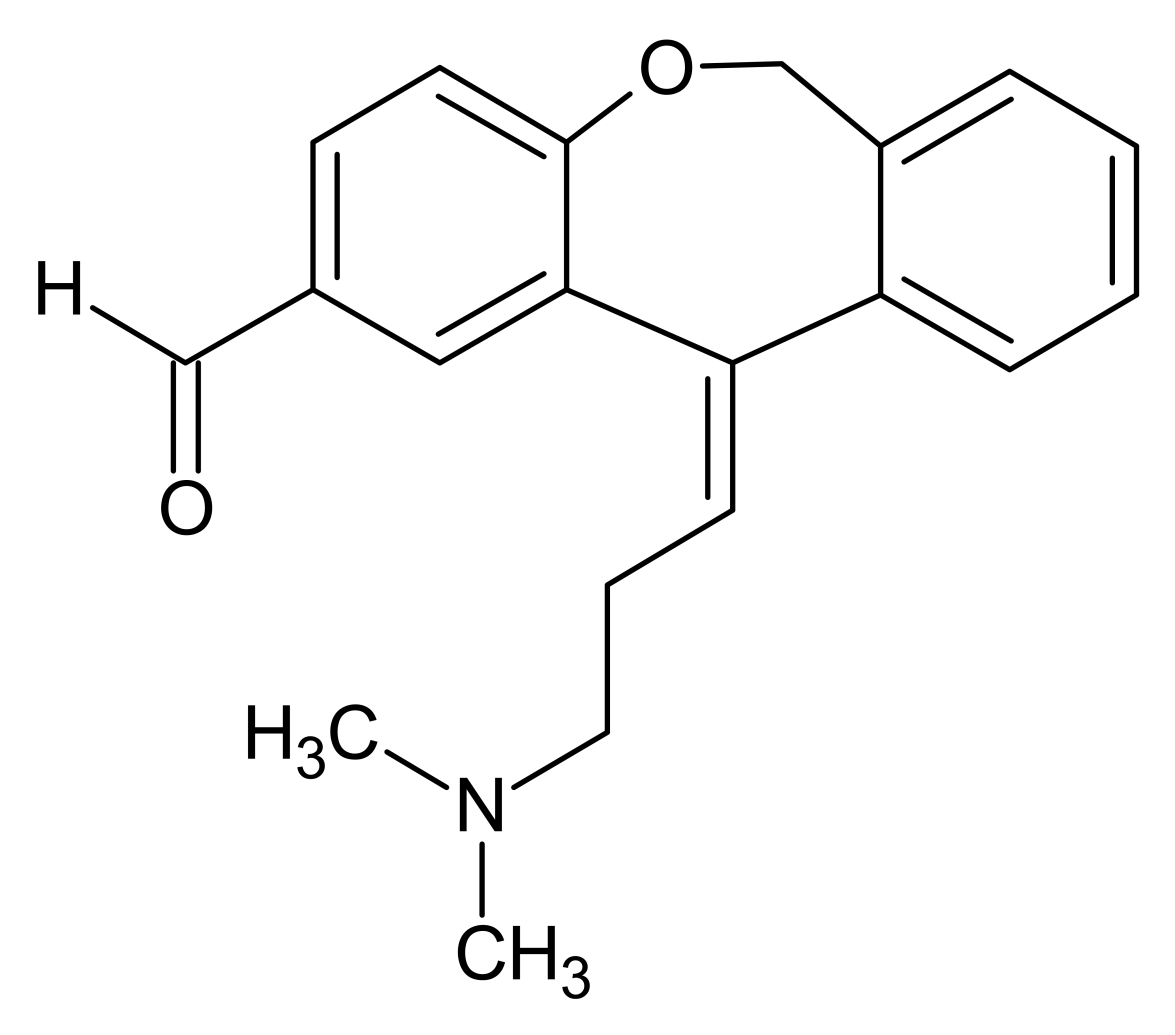	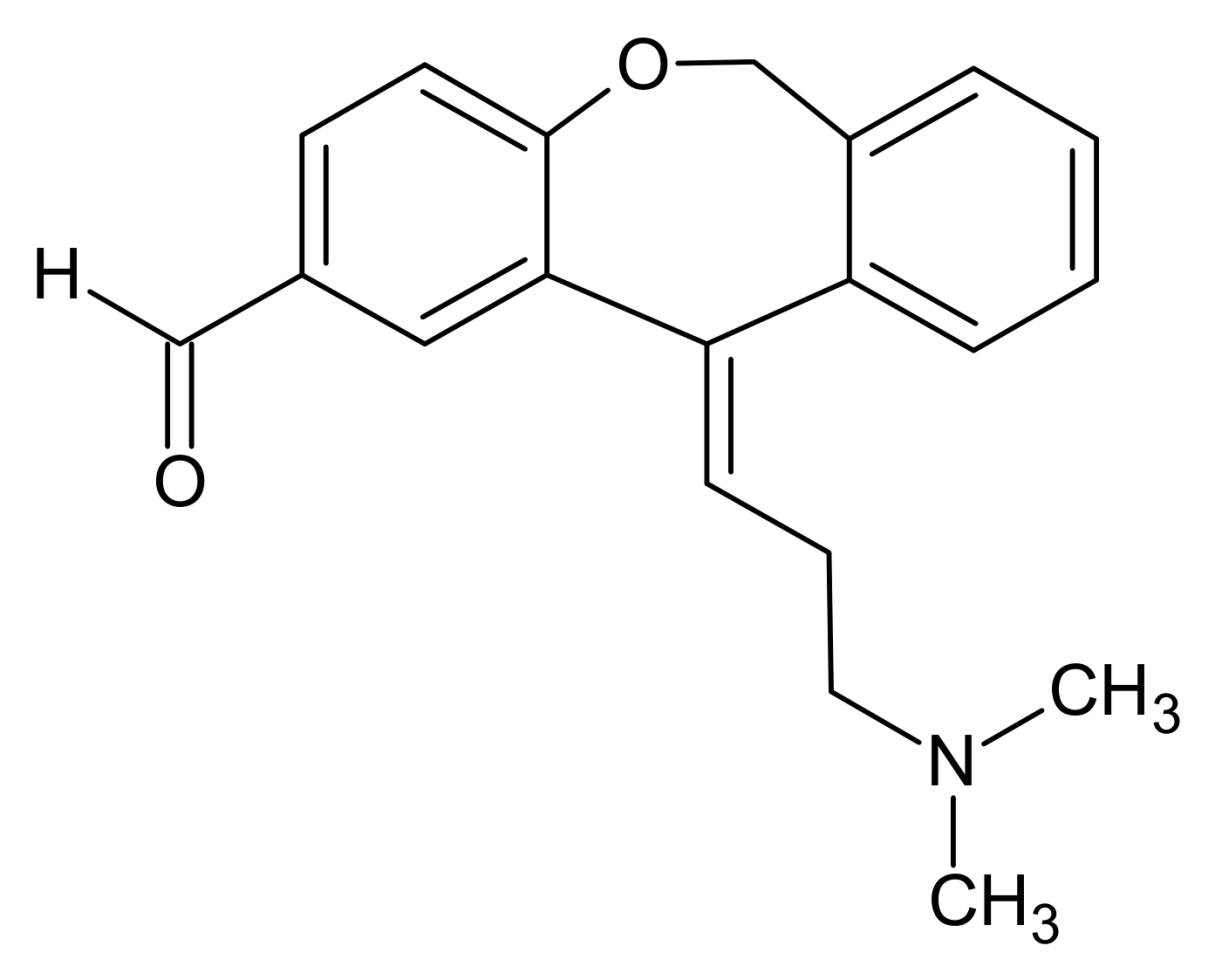	307	1, 4, 7
OLO8	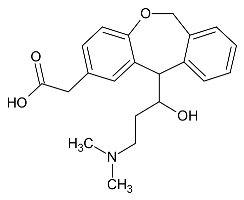		355	4, 7, 10
OLO9	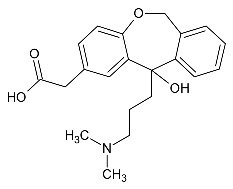		355	4, 7, 10
OLO10 (E-isomer)	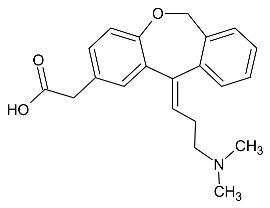		337	4, 7, 10, 13
OLO11 OLO12 (Z and E-isomer)	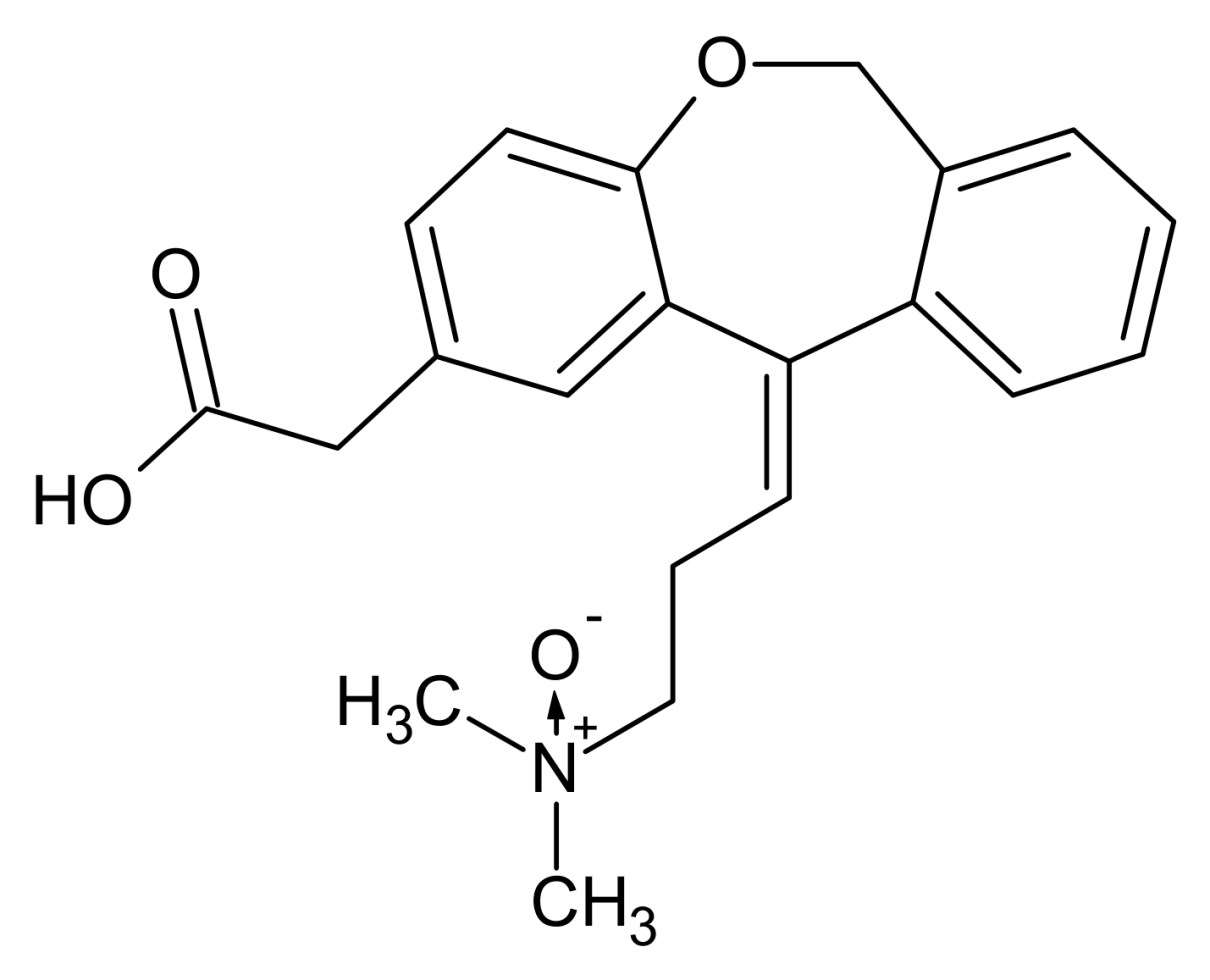	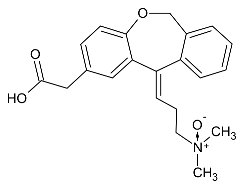	353	10, 13

## Data Availability

Data available on request due to restrictions.

## Supplementary Materials

The following supporting information can be downloaded at: https://www.mdpi.com/article/10.3390/molecules31173112/s1. The following supporting results are included: Table S1. Quantitative LC-UV assay of olopatadine (OLO); Table S2. NMR data (DMSO-d_6_, ^1^H 600 MHz and ^13^C 150 MHz) for OLO4, OLO5 and OLO10; Figure S1. ^1^H NMR spectrum of OLO1 (DMSO-d_6_, 600 MHz); Figure S2. ^1^H-^1^H COSY spectrum of OLO1 (DMSO-d_6_, 600 MHz); Figure S3. ^1^H-^13^C HSQC spectrum of OLO1 (DMSO-d_6_, ^1^H 600 MHz and ^13^C 150 MHz). The positive cross-peaks in the HSQC spectrum (blue) originate from the CH and CH_3_ groups, and the negative cross-peaks (green) from the CH_2_ groups; Figure S4. ^1^H-^13^C HMBC spectrum of OLO1 (DMSO-d_6_, ^1^H 600 MHz and ^13^C 150 MHz); Figure S5. One-dimensional selective ^1^H-^1^H ROESY spectra of OLO1 after selective refocusing of protons occurring at δ_H_ (A) 6.07 ppm (H-10), (B) 6.71 ppm (H-1) and (C) 6.91 ppm (H-3) (DMSO-d_6_, 600 MHz). Mixing time = 300 ms. Signals of source nuclei are indicated by red arrows at each spectrum; Figure S6. ^13^C NMR spectrum of OLO1 (DMSO-d_6_, 150 MHz); Figure S7. ^1^H NMR spectrum of OLO2 (DMSO-d_6_, 600 MHz); Figure S8. ^1^H-^1^H COSY spectrum of OLO2 (DMSO-d_6_, 600 MHz); Figure S9. ^1^H-^13^C HSQC spectrum of OLO2 (DMSO-d_6_, ^1^H 600 MHz and ^13^C 150 MHz). The positive cross-peaks in the HSQC spectrum (blue) originate from the CH and CH_3_ groups, and the negative cross-peaks (green) from the CH_2_ groups; Figure S10. ^1^H-^13^C HMBC spectrum of OLO2 (DMSO-d_6_, ^1^H 600 MHz and ^13^C 150 MHz); Figure S11. One-dimensional selective ^1^H-^1^H ROESY spectra of OLO2 after selective refocusing of protons occurring at δ_H_ (A) 5.67 ppm (H-10), (B) 6.83 ppm (H-1) and (C) 6.99 ppm (H-3) (DMSO-d_6_, 600 MHz). Mixing time = 300 ms. Signals of source nuclei are indicated by red arrows at each spectrum; Figure S12. ^13^C NMR spectrum of OLO2 (DMSO-d_6_, 150 MHz); Figure S13. ^1^H NMR spectrum of OLO3 (DMSO-d_6_, 600 MHz); Figure S14. ^1^H-^1^H COSY spectrum of OLO3 (DMSO-d_6_, 600 MHz); Figure S15. ^1^H-^13^C HSQC spectrum of OLO3 (DMSO-d_6_, ^1^H 600 MHz and ^13^C 150 MHz). The positive cross-peaks in the HSQC spectrum (blue) originate from the CH and CH_3_ groups, and the negative cross-peaks (green) from the CH_2_ groups; Figure S16. ^1^H-^13^C HMBC spectrum of OLO3 (DMSO-d_6_, ^1^H 600 MHz and ^13^C 150 MHz) with delay for evolution of long range couplings [1/(2*J_Long range_)] of 61.25 ms (corresponding to J_Long range_ = 8 Hz); Figure S17. ^1^H-^13^C HMBC spectrum of OLO3 (DMSO-d_6_, ^1^H 600 MHz and ^13^C 150 MHz) with delay for evolution of long range couplings [1/(2*J_Long range_)] of 125 ms (corresponding to J_Long range_ = 4 Hz); Figure S18. One-dimensional selective ^1^H-^1^H ROESY spectra of OLO3 after selective refocusing of protons occurring at δ_H_ (A) 5.72 ppm (H-10), (B) 6.97 ppm (H-3) and (C) 4.14 ppm (H-20) (DMSO-d_6_, 600 MHz). Mixing time = 300 ms. Signals of source nuclei are indicated by red arrows at each spectrum; Figure S19. ^13^C NMR spectrum of OLO3 (DMSO-d_6_, 150 MHz); Figure S20. The structures of OLO4, OLO5 and OLO6.

## References

[1] https://www.drugbank.com.

[2] Derayea S.M., Badr El-Din K.M., Ahmed A.S., Ahmed K.A., Oraby M. (2024). Determination of antihistaminic drugs alcaftadine and olopatadine hydrochloride via ion-pairing with eosin Y as a spectrofluorimetric and spectrophotometric probe: Application to dosage forms. BMC Chem..

[3] Bosch J., Bachs J., Gómez A.M., Griera R., Écija M., Amat M. (2012). Stereoselective syntheses of the antihistaminic drug olopatadine and its E-isomer. J. Org. Chem..

[4] Pan C., Liu F., Ji Q., Wang W., Drinkwater D., Vivilecchia R. (2006). The use of LC/MS, GC/MS, and LC/NMR hyphenated techniques to identify a drug degradation product in pharmaceutical development. J. Pharm. Biomed. Anal..

[5] Bhangare D., Rajput N., Jadav T., Kumar Sahu A.K., Tekade R.K., Sengupta P. (2022). Systematic strategies for degradation kinetic study of pharmaceuticals: An issue of utmost importance concerning current stability analysis practices. J. Anal. Sci. Technol..

[6] Sahu R.K., Singh B., Saraf S.A., Kaithwas G., Kishor K. (2014). Photochemical toxicity of drugs intended for ocular use. Arh. Hig. Rada Toksikol..

[7] (1998). Photostability Testing of New Active Substances and Medicinal Products.

[8] Yadav A., Yadav M., Mishra S. (2023). A review on drug stability. Int. J. Sci. Res. Arch..

[9] Kleinman M.H., Baertschi S.W., Alsante K.M., Reid D.L., Mowery M.D., Shimanovich R., Ott M.A. (2014). In silico prediction of pharmaceutical degradation pathways: A bench marking study. Mol. Pharm..

[10] Basnival P.K., Jain D. (2019). Intrinsic stability study and forced degradation profiling of olopatadine hydrochloride by RPHPLC-DAD-HRMS method. Turk. J. Pharm. Sci..

[11] Jurkic M.L., Buratovic M., Valentic I., Stanfel D. (2014). UHPLC study on the degradation profiles of olopatadine hydrochloride in eye drops subjected to heat and filtration methods of sterilisation. Chromatographia.

[12] Kowtharapu L.P., Katari N.K., Sandoval C.A., Muchakayala S.K., Rekulapally V.K. (2022). Green liquid chromatography method for the determination of related substances present in olopatadine HCl nasal spray formulation, robustness by design expert. J. AOAC Int..

[13] Mahajan A.A., Mohanraj K., Kale S., Thaker A.K. (2013). Study of olopatadine hydrochloride under ICH recommended stress conditions by LC, LC-MS/TOF for identification, and characterization of degradation products. J. Liq. Chromatogr. Rel. Technol..

[14] Jadhav S., Gosar A., Jadkar A., Ankam R., Dhatrak C. (2019). Isolation and characterization photodegradation impurities of drug product olopatadine hydrochloride by spectral techniques. Chem. Biomol. Eng..

[15] (2013). Guidance on Photosafety Evaluation of Pharmaceuticals.

[16] Kounaris Fuziki M.E., Ribas L.S., Tusset A.M., Brackmann R., Dos Santos O.A.A., Goncalves Lenzi G. (2023). Pharmaceutical compounds photolysis: pH influence. Heliyon.

[17] (2005). ICH.

[18] Upmanyu N., Shah K., Porwal P.K., Talele G.S. (2016). Degradation kinetics of olopatadine HCl using a validated UV-area under curve method. J. Anal. Pharm. Res..

[19] Jang D.-J., Lee J.H., Kim D.H., Kim J.-W., Koo T.-S., Cho K.H. (2023). The development of super saturated rebamipide eye drops for enhanced solubility, stability, patient compliance and bioavailability. Pharmaceutics.

[20] (2003). Stability Testing of New Active Substances and Products.

[21] Claridge T.D.W. (2016). High-Resolution NMR Techniques in Organic Chemistry.

[22] Bain A.D. (2003). Chemical exchange in NMR. Prog. Nucl. Magn. Reson. Spectrosc..

[23] Silverstein R.M., Webster F.X., Kiemle D.J., Bryce D.L. (2014). Spectrometric Identification of Organic Compounds.

[24] Walaszek K., Ciach T., Markowicz-Piasecka M. (2023). Trends development and quality assessment of pharmaceutical formulations F2ɑanalogues in the glaucoma treatment. Eur. J. Pharm. Sci..

[25] OSIRIS Property Explorer. https://www.organic-chemistry.org/prog/peo/tox.html.

[26] Toxtree. https://toxtree.sourceforge.net.

[27] Heleno Ferreira R.B., Duarte J.A., Ferreira F.D., Souza de Oliveira L.F., Mansur Machado M., Donadel Malesuik M., Reisdorfer Paula F., Steppe M., Shermann Schapoval E.E., Soldateli Paim C. (2019). Biological safety studies and simulateneous determination of linagliptin and synthetic impurities by LC-PDA. J. Anal. Methods Chem..

[28] Roberts D.W., Aptula A., Schultz T.W., Shen J., Api A.M., Bhatia S., Kromidas L. (2015). A practical guidance for Cramer class determination. Regul. Toxicol. Pharmacol..

[29] Api A.M., Belsito D., Bruze M., Cadby P., Calow P., Dagli M.L., Dekant W., Ellis G., Fryer A.D., Fukayama M. (2016). Criteria for the Research Institute for Fragrance Materials, Inc. (RIFM) safety evaluation process for fragrance ingredients. Food Chem. Toxicol..

[30] More S.J., Bampidis V., Benford D., Bragard C., Halldorsson T.I., Hernandez-Jerez A.F., Hougaard B.S., Koutsoumanis K.P., Machera K., EFSA Scientific Committee (2019). Guidance on the use of the Threshold of Toxicological Concern approach in food safety assessment. EFSA J..

[31] U.S. Food and Drug Administration (2023). Quality Considerations for Topical Ophthalmic Drug Products: Guidance for Industry. https://www.fda.gov/regulatory-information/search-fda-guidance-documents/quality-considerations-topical-ophthalmic-drug-products.

