# Supplementary material for: Comprehensive Analytical Investigation of the Photodegradation of Olopatadine and In Silico Toxicity Assessment of Its Photodegradation Products
Source: Molecules. 2026 Sep 4;31(17):3112. doi: 10.3390/molecules31173112 (PMC13567034; doi:10.3390/molecules31173112)
Supplement: Supplementary file 1 [file molecules-31-03112-s001.zip › molecules-4517201-supplementary.pdf]

Table S1. Quantitative LC-UV assay of olopatadine (OLO).

**Parameter**	
Linearity range (µg/ml)	10–100
Slope (mean ± SD, n=5)	8768 ± 14.863
Intercept (mean ± SD, n=5)	- 16800 ± 556.54
R^2 (mean ± SD, n=5)	0.9999±0.0001
LOD (µg/ml)	0.21
LOQ (µg/ml)	0.63
% Recovery (mean ± SD, n=9)	98.79 ± 0.82
% RSD for within-day precision (n=3)	0.93
% RSD for between-day precision (n=9)	3.27

SD=standard deviation; RSD=relative standard deviation.

Table S2. NMR data (DMSO-d_6, ^{1}H 600 MHz and ^{13}C 150 MHz) for OLO4, OLO5 and OLO10.

	**OLO4**		**OLO5**		**OLO10**	
**No.**	**^{1}H**	**^{13}C**	**^{1}H**	**^{13}C**	**^{1}H**	**^{13}C**
1	7.08, m[a]	132.5	7.18, d (2.3)	131.3	7.15, d (1.9)	131.2
2	-	126.7	-	126.9	-	128.3
3	7.08, m[a]	130.8	7.04, dd (8.3, 2.3)	130.5	7.02, dd (8.3, 2.0)	130.4
4	6.78. d (9.0)	119.7	6.68, d (8.3)	119.3	6.66, d (8.3)	119.2
5	-	154.4	-	154.0	-	153.8
6	-	123.6	-	126.9	-	127.0
7	3.63, s	39.5	3.60, s	39.5	3.46, s	40.5
8	-	172.2	-	172.1	-	173.5
9	-	139.8	-	139.6	-	139.2
10	5.67, t (7.1)	130.4	6.00, t (7.4)	129.8	6.01, t (7.4)	130.7
11	2.58, q (7.1)	26.9	2.30, brm	26.7	2.25, m	27.4
12	2.68, t (7.0)	58.0	2.61, brm	57.9	2.39, m	58.7
13	2.31, s	44.3	2.22, s	44.2	2.09, s	45.1
14	-	145.6	-	140.9	-	141.2
15	7.26, dd (7.6, 1.0)	126.3	7.26, dd (7.5, 1.3)	128.1	7.26, dd (7.4, 1.1)	128.1
16	7.38, m[b]	128.3	7.41, td (7.5, 1.5)	129.1	7.40, td (7.5, 1.4)	130.0
17	7.32, td (7.3, 1.3)	128.1	7.36, td (7.4, 1.5)	129.0	7.36, td (7.4, 1.4)	128.3
18	7.37, m[b]	129.6	7.49, dd (7.4, 1.4)	128.9	7.49, dd (7.3, 1.2)	128.8
19	-	134.0	-	134.6	-	134.6
20	5.17, brs	70.0	4.86, brs<br>5.44, brs	69.5	4.88, brs<br>5.41, brs	69.5
21	3.60, s	52.1	3.61, s	52.1	-	-

[a], [b] Overlapping multiplets.

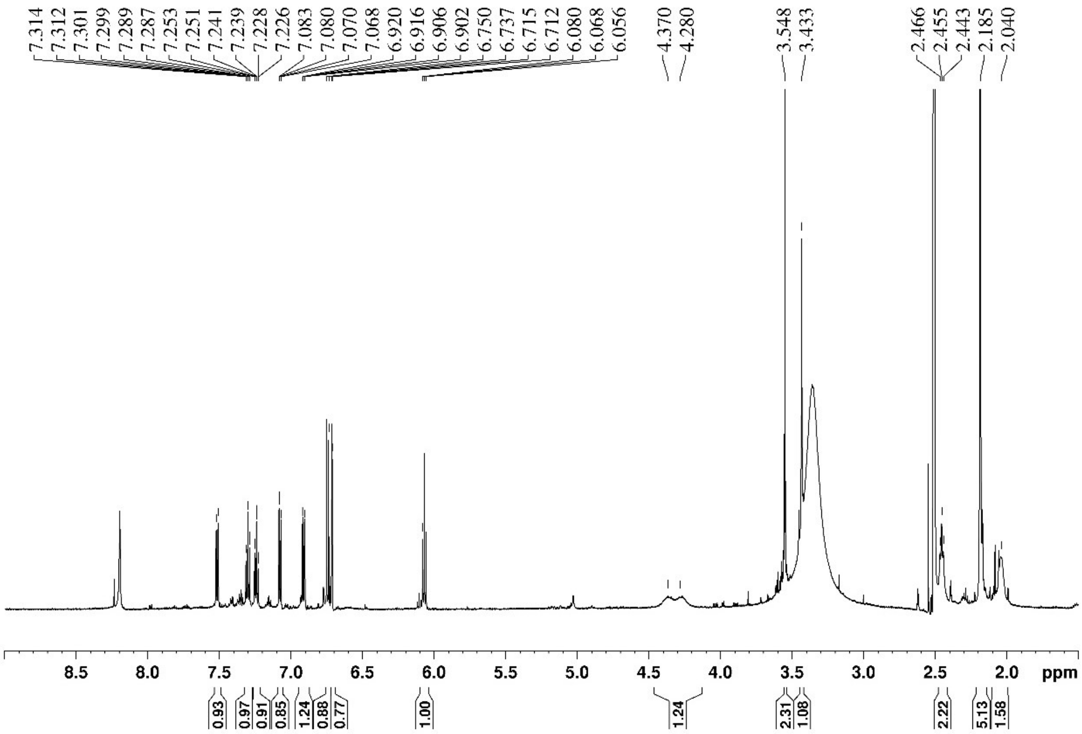

Figure S1. ^{1}H NMR spectrum of OLO1 (DMSO-d_6, 600 MHz).

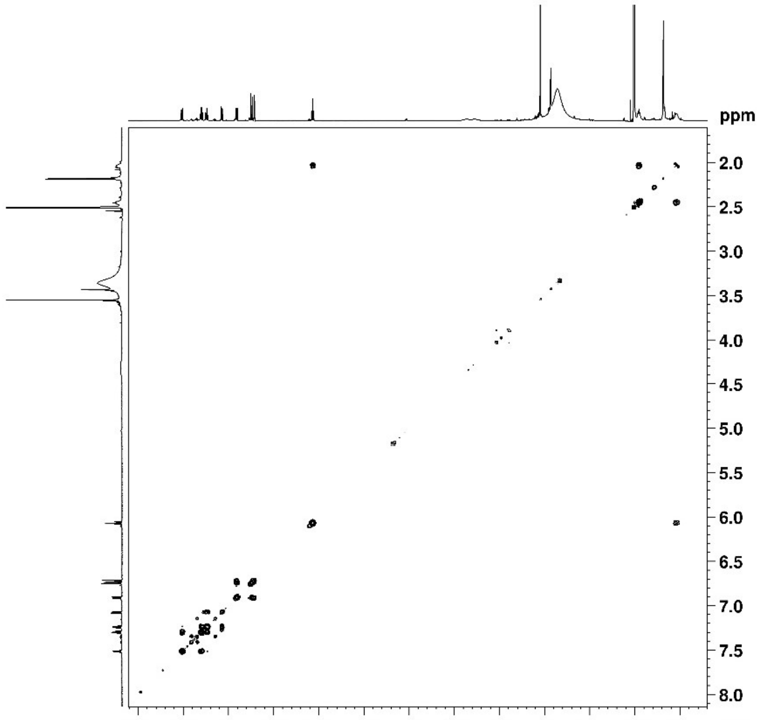

Figure S2. ^{1}H-^{1}H COSY spectrum of OLO1 (DMSO-d_6, 600 MHz).

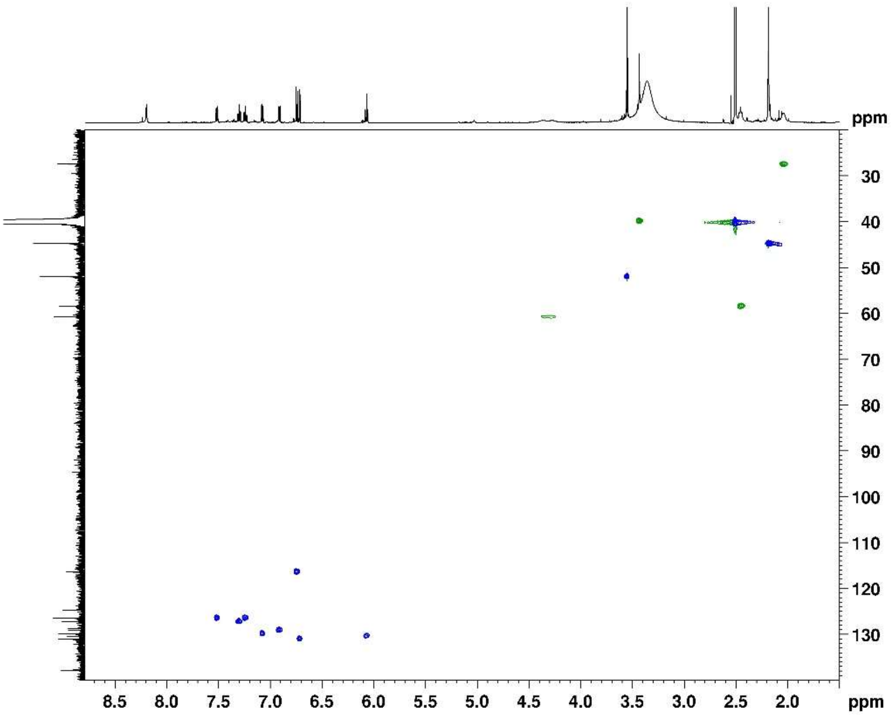

Figure S3. ^{1}H-^{13}C HSQC spectrum of OLO1 (DMSO-d_6, ^{1}H 600 MHz and ^{13}C 150 MHz). The positive cross-peaks in the HSQC spectrum (blue) originate from the CH and CH_3 groups, and the negative cross-peaks (green) from the CH_2 groups.

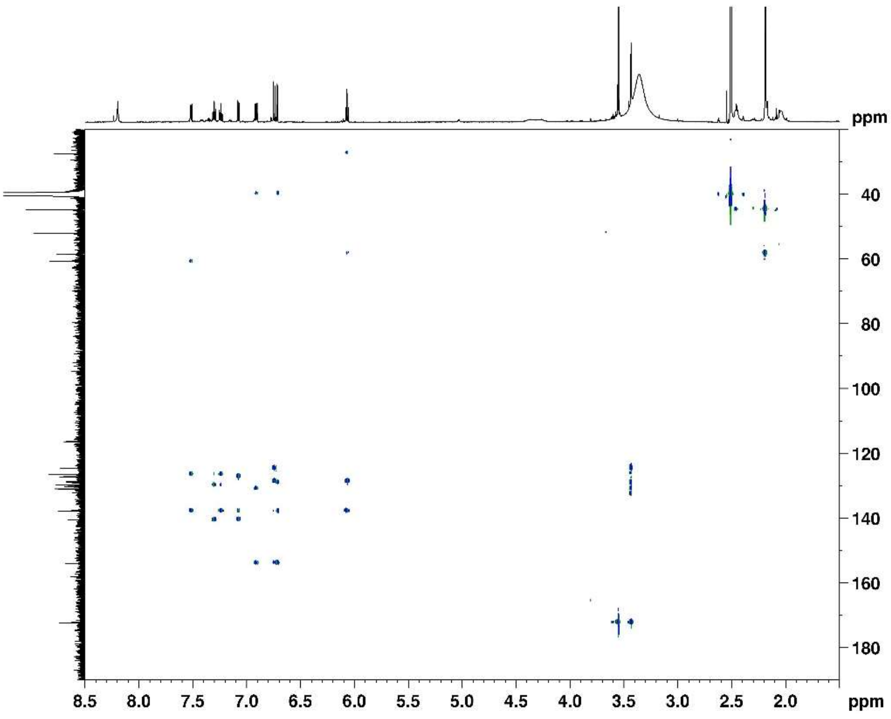

Figure S4. ^{1}H-^{13}C HMBC spectrum of OLO1 (DMSO-d_6, ^{1}H 600 MHz and ^{13}C 150 MHz).

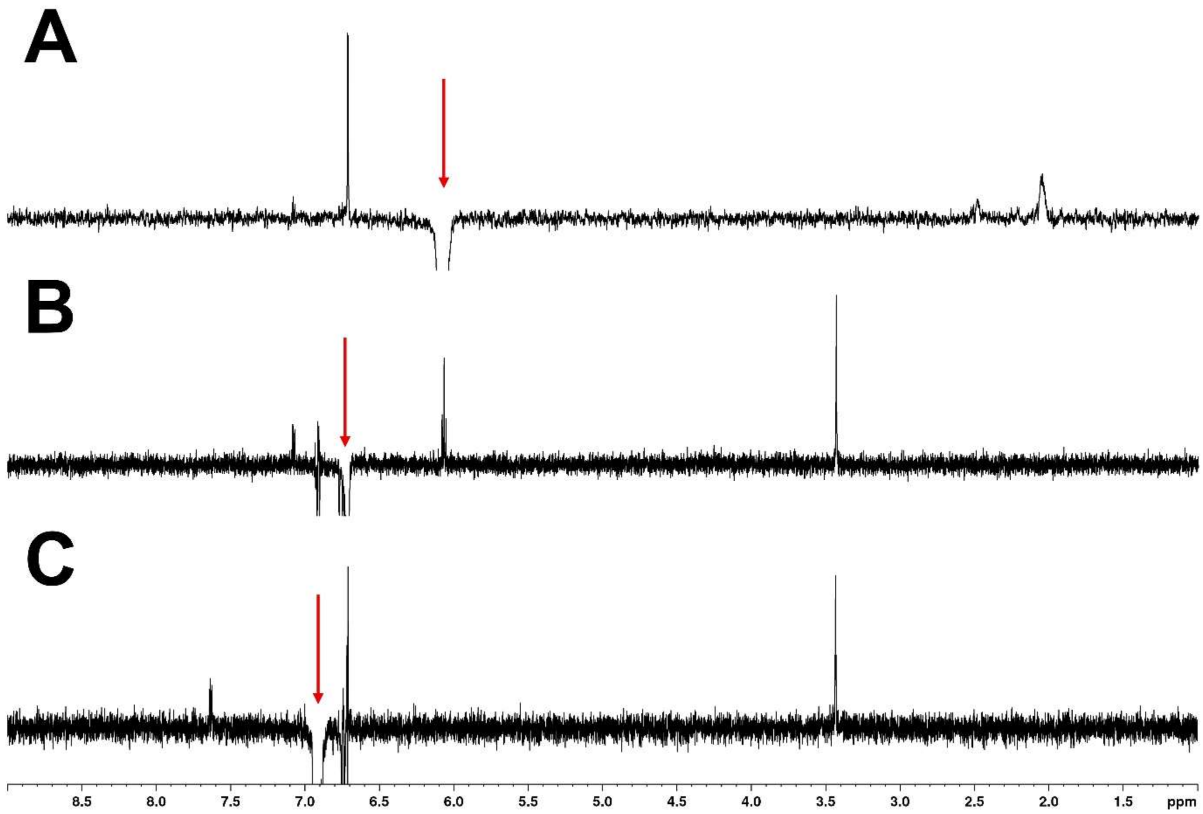

Figure S5. 1D selective ^{1}H-^{1}H ROESY spectra of OLO1 after selective refocusing of protons occurring at δ_H A) 6.07 ppm (H-10), B) 6.71 ppm (H-1) and C) 6.91 ppm (H-3) (DMSO-d_6, 600 MHz). Mixing time = 300 ms. Signals of source nuclei are indicated by red arrows at each spectrum.

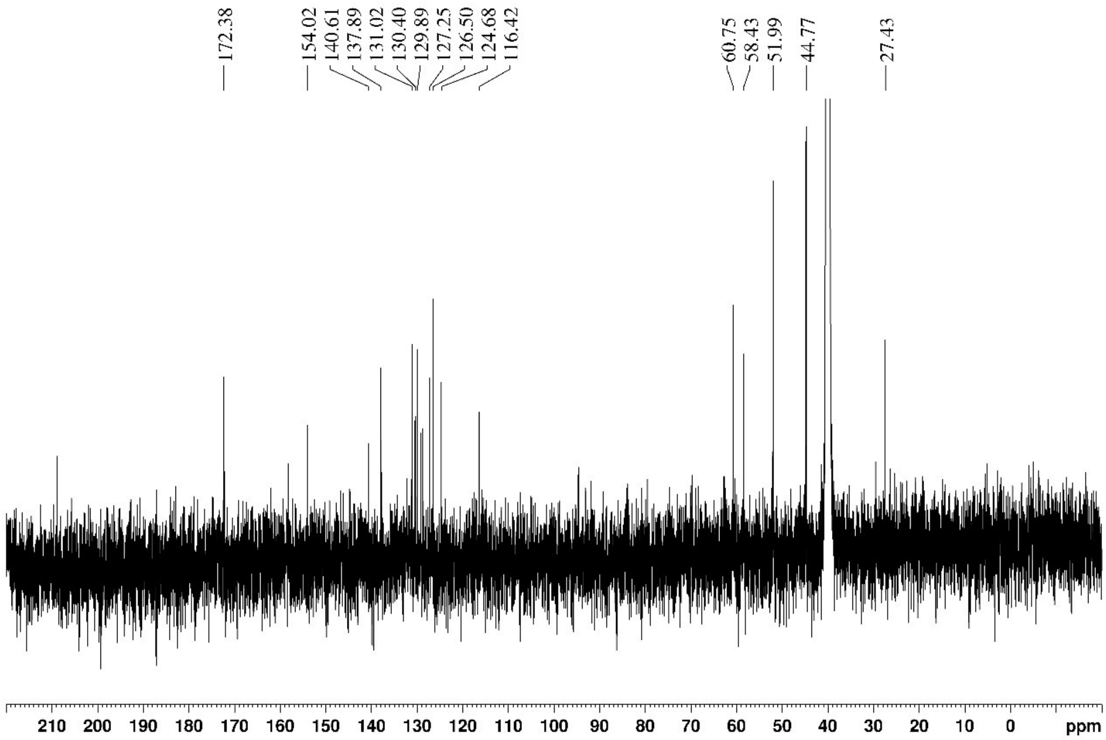

Figure S6. ^{13}C NMR spectrum of OLO1 (DMSO-d_6, 150 MHz).

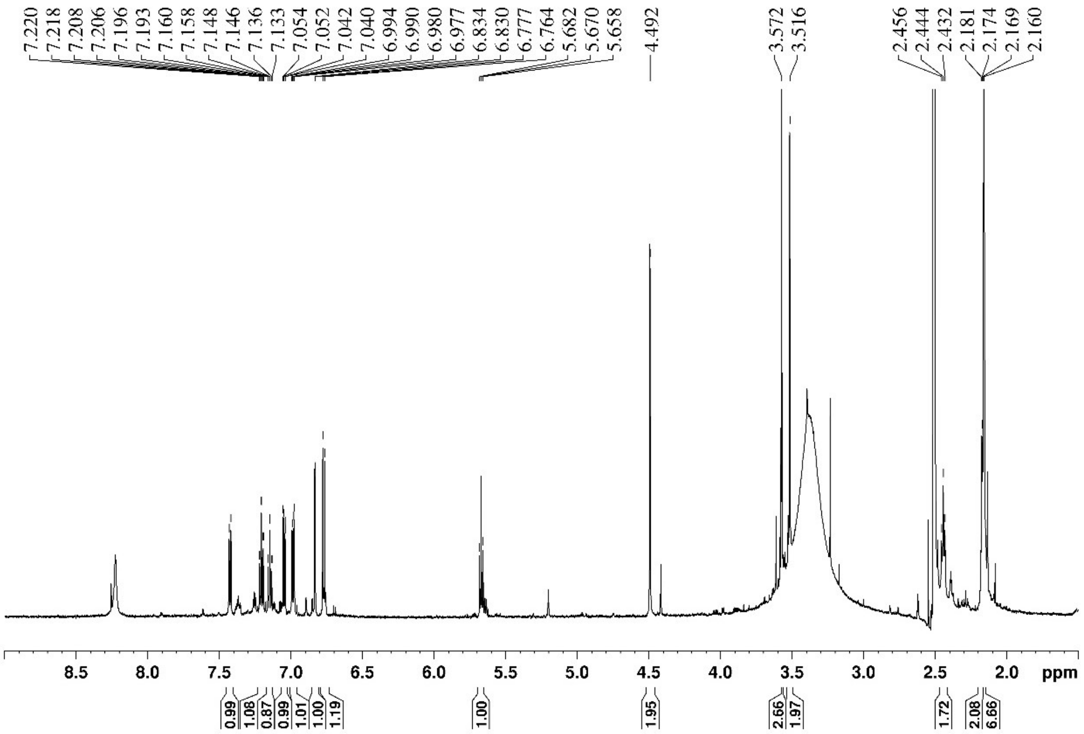

Figure S7. ^{1}H NMR spectrum of OLO2 (DMSO-d_6, 600 MHz).

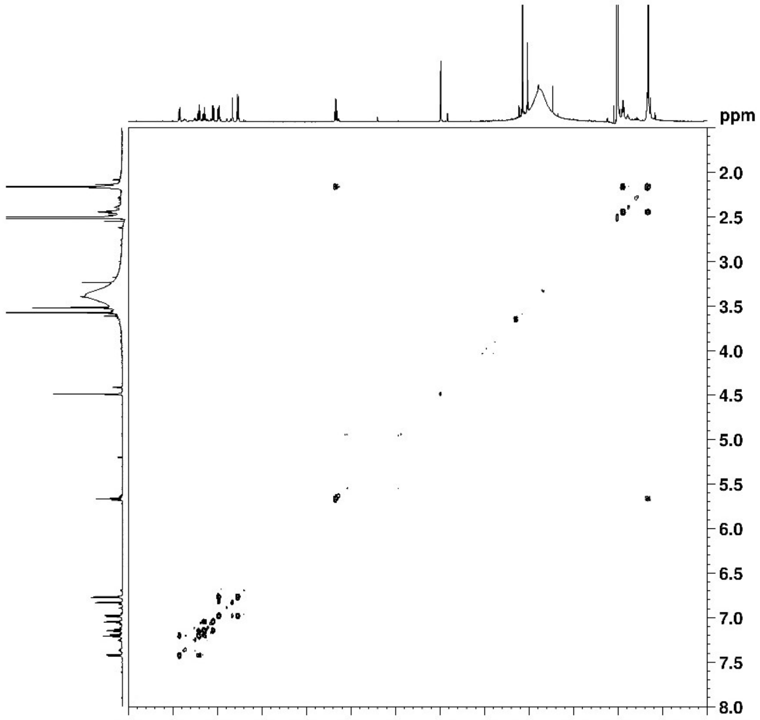

Figure S8. ^{1}H-^{1}H COSY spectrum of OLO2 (DMSO-d_6, 600 MHz).

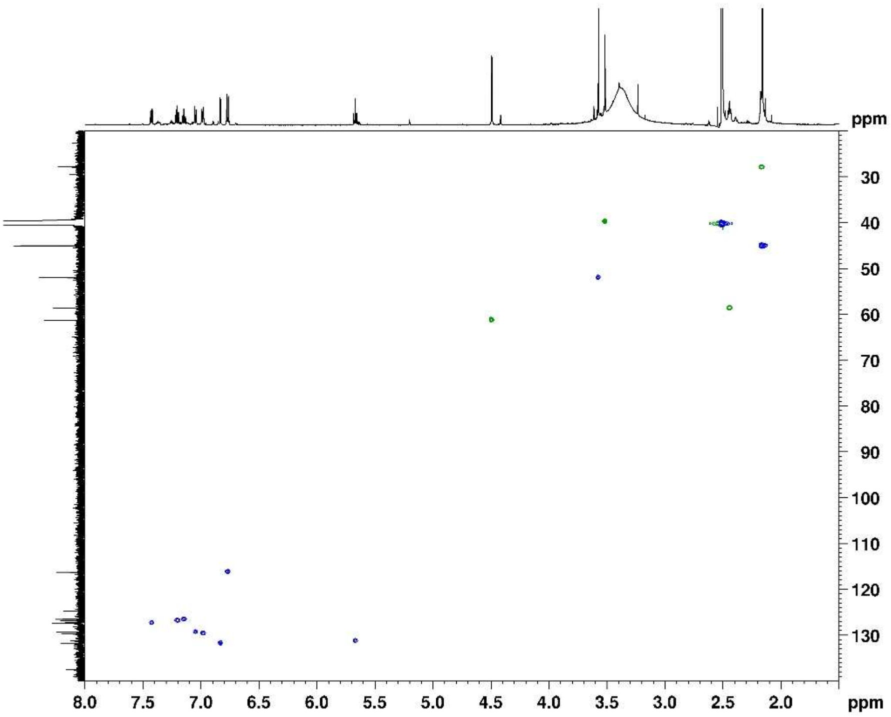

Figure S9. ^{1}H-^{13}C HSQC spectrum of OLO2 (DMSO-d_6, ^{1}H 600 MHz and ^{13}C 150 MHz). The positive cross-peaks in the HSQC spectrum (blue) originate from the CH and CH_3 groups, and the negative cross-peaks (green) from the CH_2 groups.

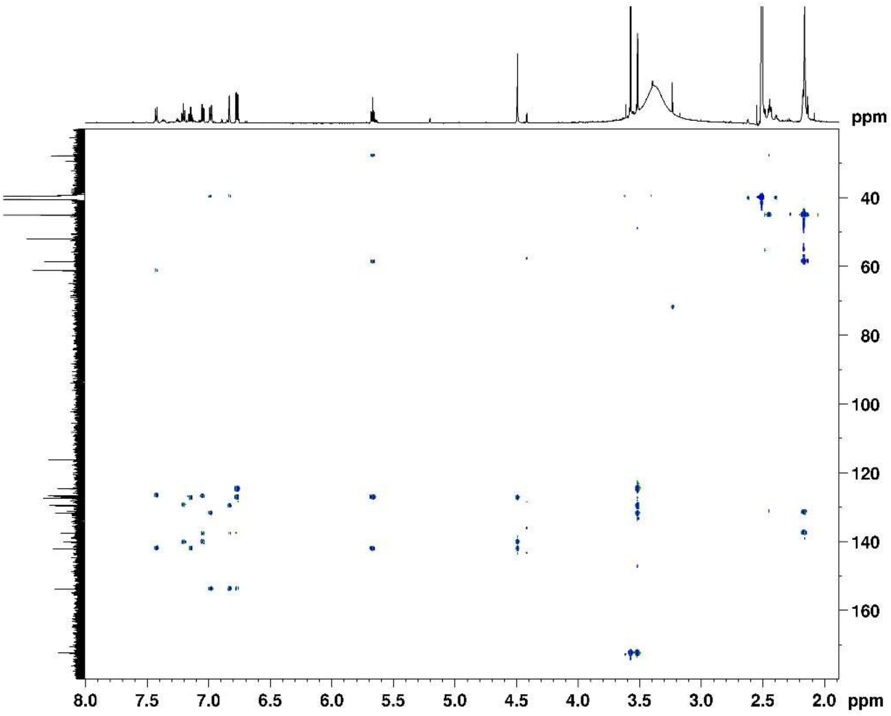

Figure S10. ^{1}H-^{13}C HMBC spectrum of OLO2 (DMSO-d_6, ^{1}H 600 MHz and ^{13}C 150 MHz).

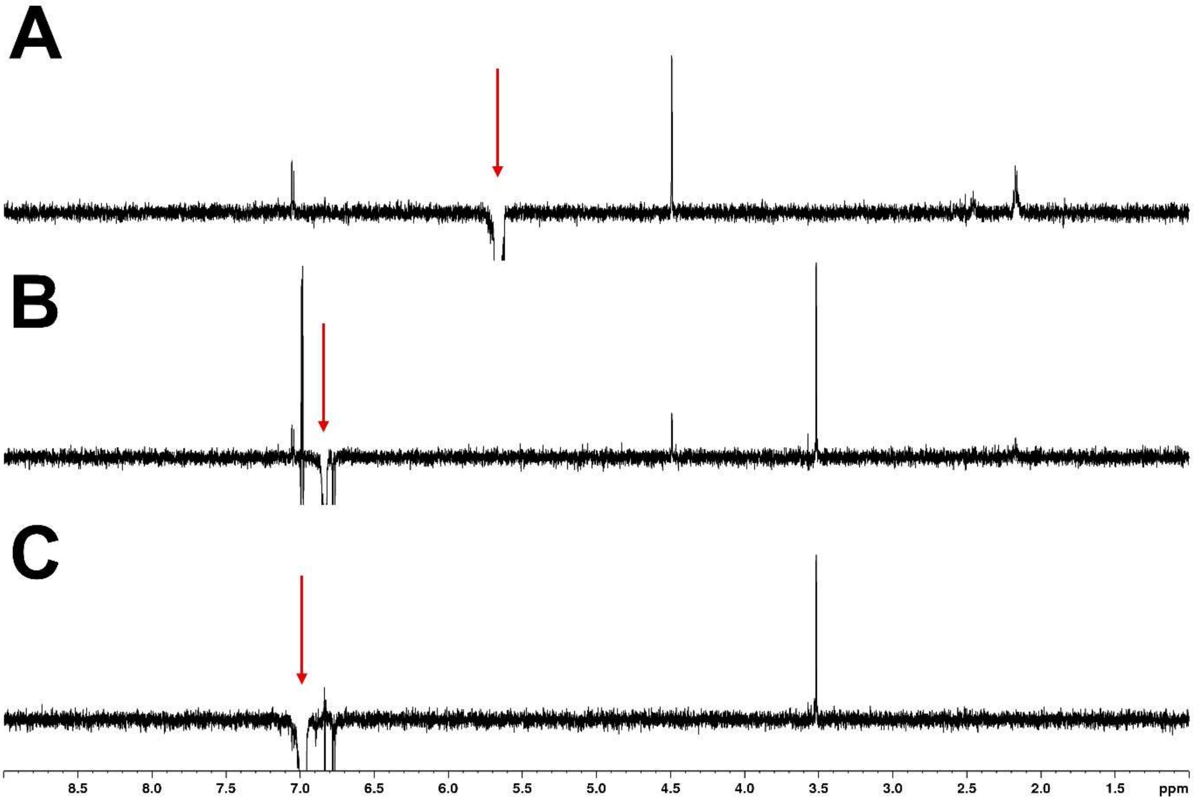

Figure S11. 1D selective ^{1}H-^{1}H ROESY spectra of OLO2 after selective refocusing of protons occurring at δ_H A) 5.67 ppm (H-10), B) 6.83 ppm (H-1) and C) 6.99 ppm (H-3) (DMSO-d_6, 600 MHz). Mixing time = 300 ms. Signals of source nuclei are indicated by red arrows at each spectrum.

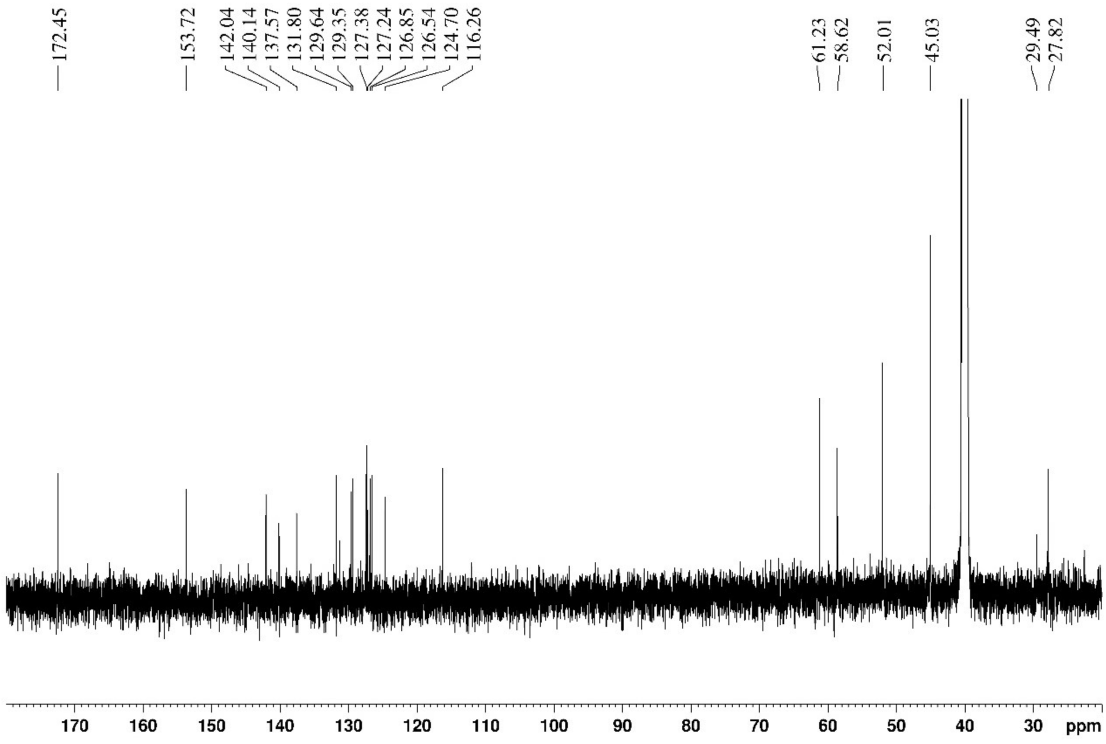

Figure S12. ^{13}C NMR spectrum of OLO2 (DMSO-d_6, 150 MHz).

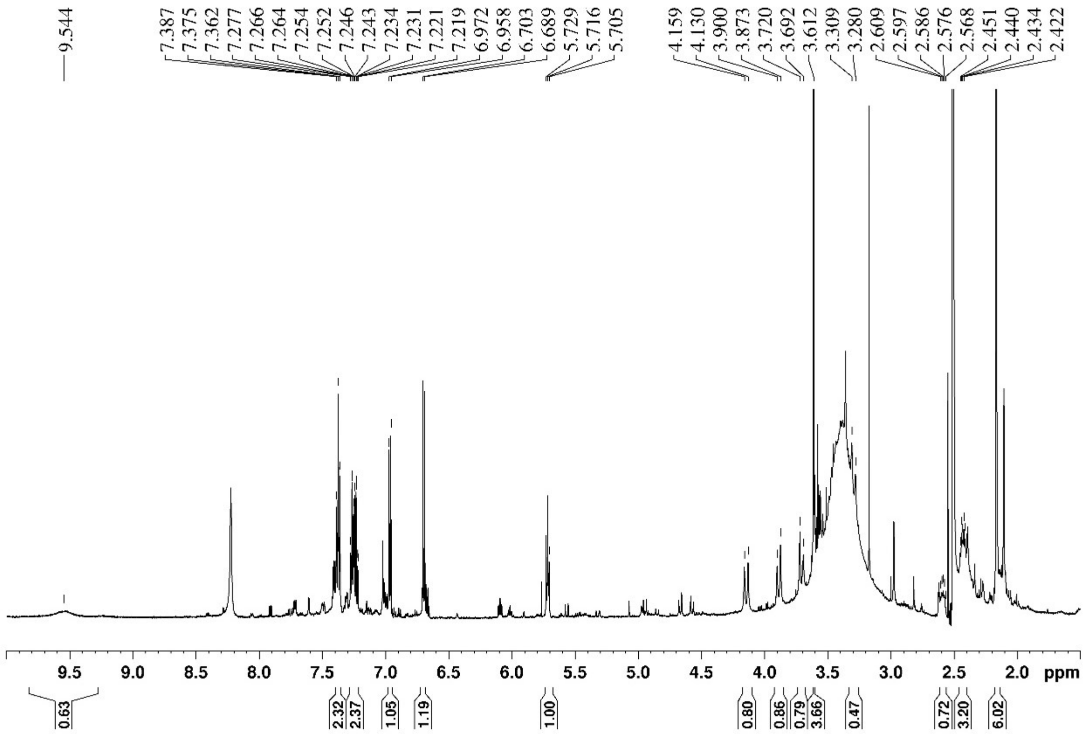

Figure S13. ^{1}H NMR spectrum of OLO3 (DMSO-d_6, 600 MHz).

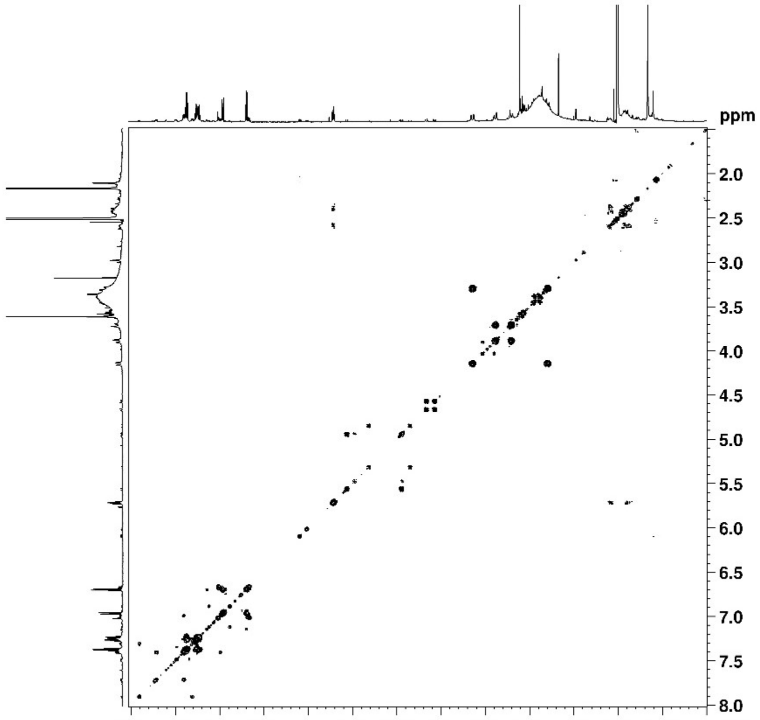

Figure S14. ^{1}H-^{1}H COSY spectrum of OLO3 (DMSO-d_6, 600 MHz).

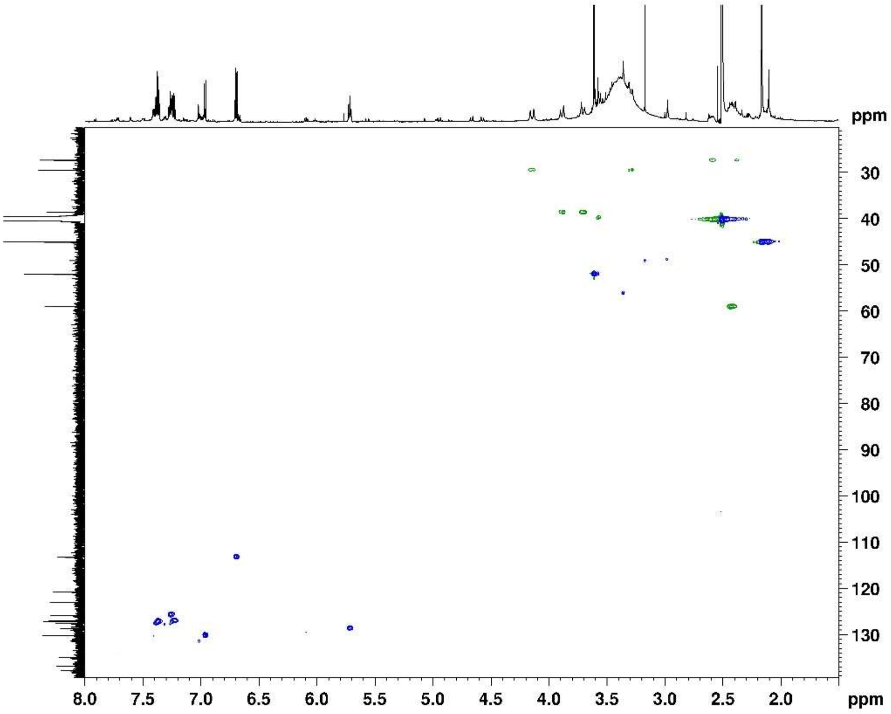

Figure S15. ^1H-^{13}C HSQC spectrum of OLO3 (DMSO-d_6, ^1H 600 MHz and ^{13}C 150 MHz). The positive cross-peaks in the HSQC spectrum (blue) originate from the CH and CH_3 groups, and the negative cross-peaks (green) from the CH_2 groups.

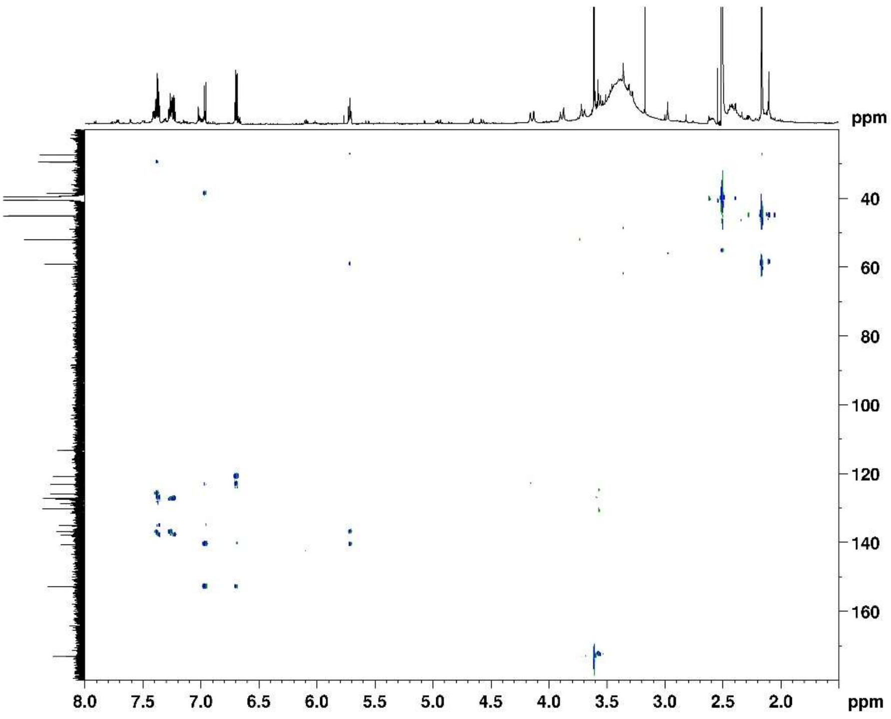

Figure S16. ^1H-^{13}C HMBC spectrum of OLO3 (DMSO-d_6, ^1H 600 MHz and ^{13}C 150 MHz) with delay for evolution of long range couplings [1/(2*$J_{Long\ range}$)] of 61.25 ms (corresponding to $J_{Long\ range}$= 8 Hz).

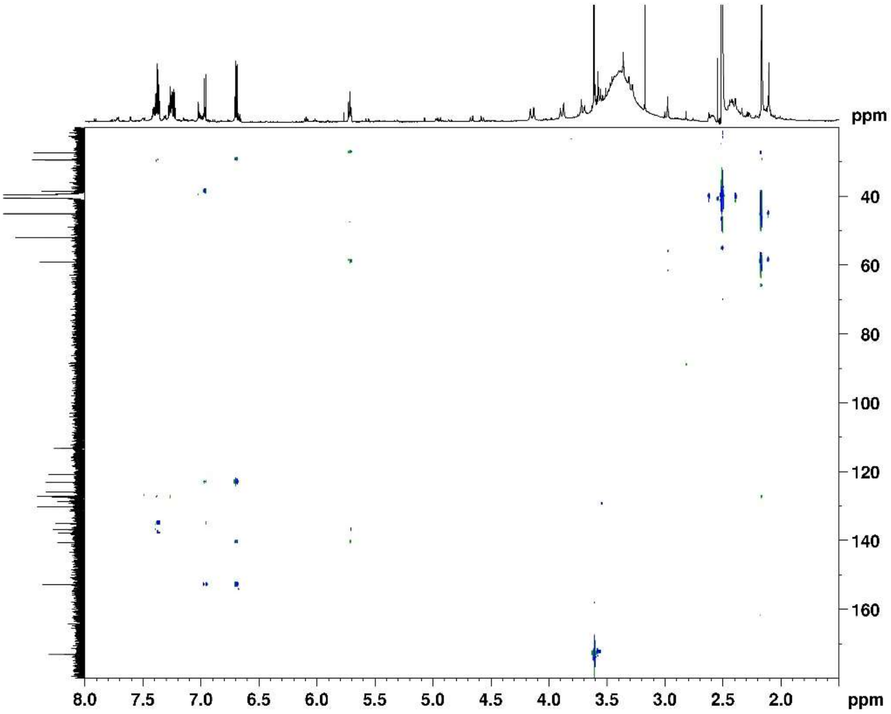

Figure S17. ^{1}H-^{13}C HMBC spectrum of OLO3 (DMSO-d_6, ^{1}H 600 MHz and ^{13}C 150 MHz) with delay for evolution of long range couplings [$1/(2{*}J_{Long\ range})$] of 125 ms (corresponding to $J_{Long\ range}$= 4 Hz).

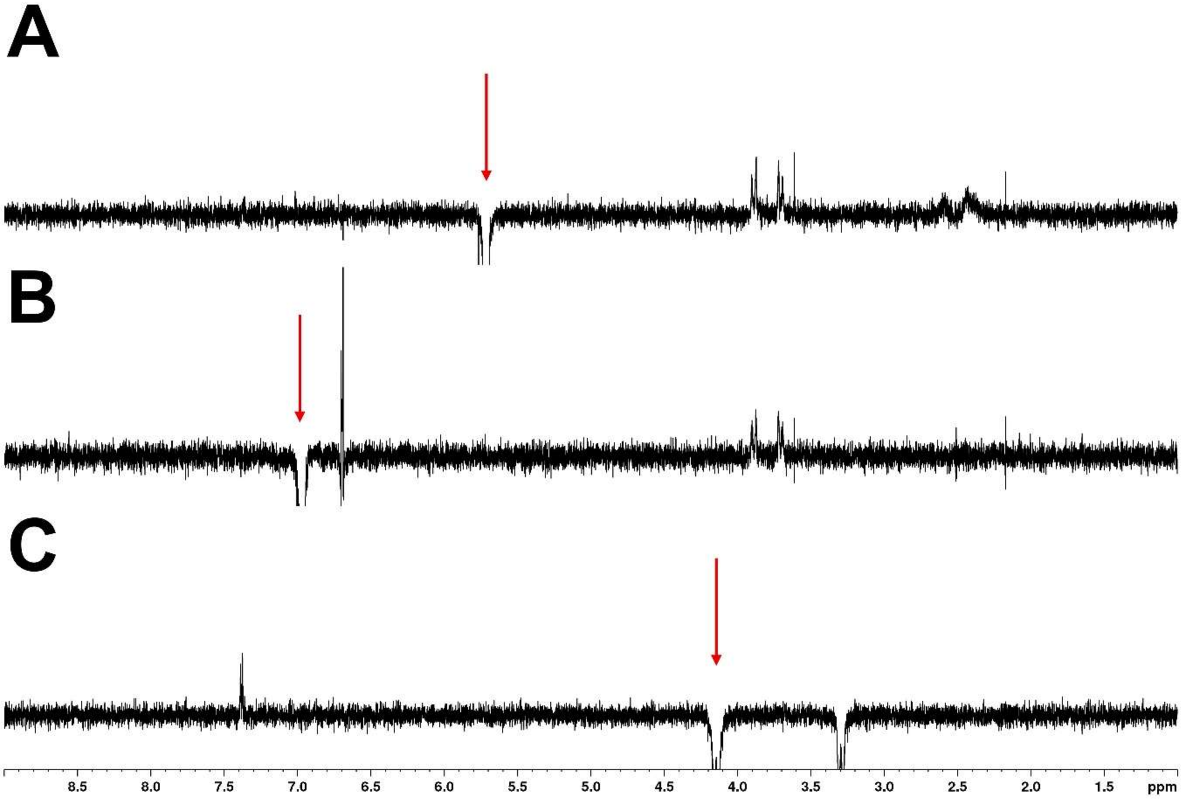

Figure S18. 1D selective ^{1}H-^{1}H ROESY spectra of OLO3 after selective refocusing of protons occurring at δ_H A) 5.72 ppm (H-10), B) 6.97 ppm (H-3) and C) 4.14 ppm (H-20) (DMSO-d_6, 600 MHz). Mixing time = 300 ms. Signals of source nuclei are indicated by red arrows at each spectrum.

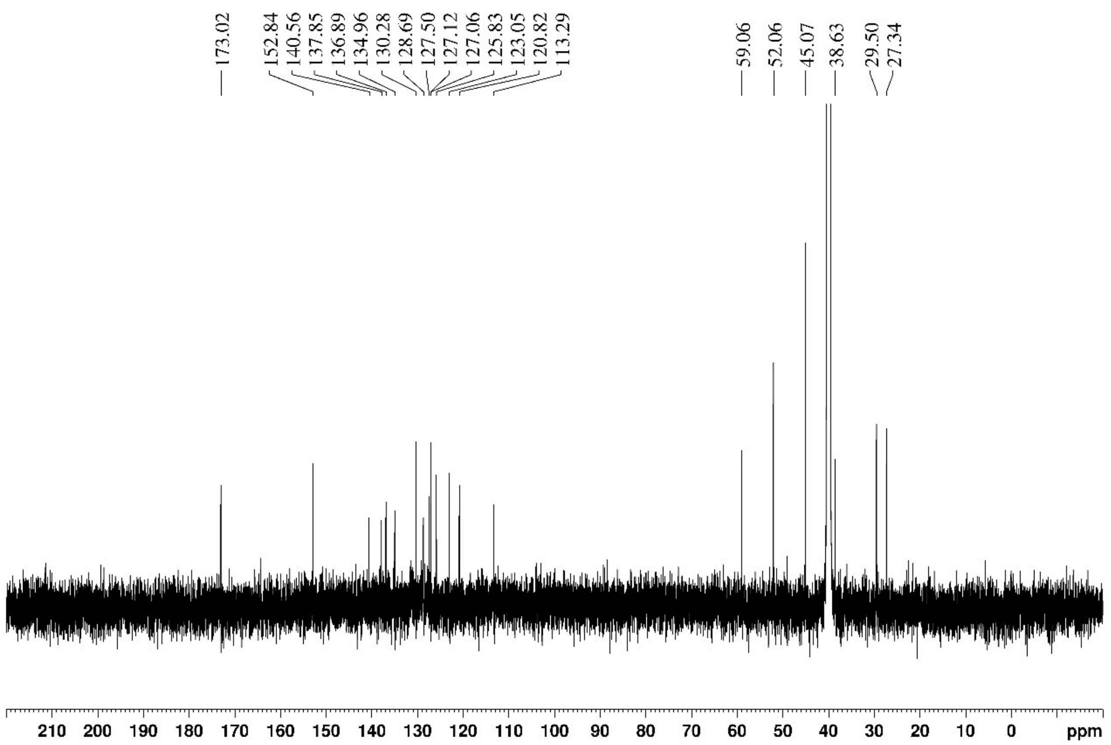

Figure S19. ^{13}C NMR spectrum of OLO3 (DMSO-d_6, 150 MHz).

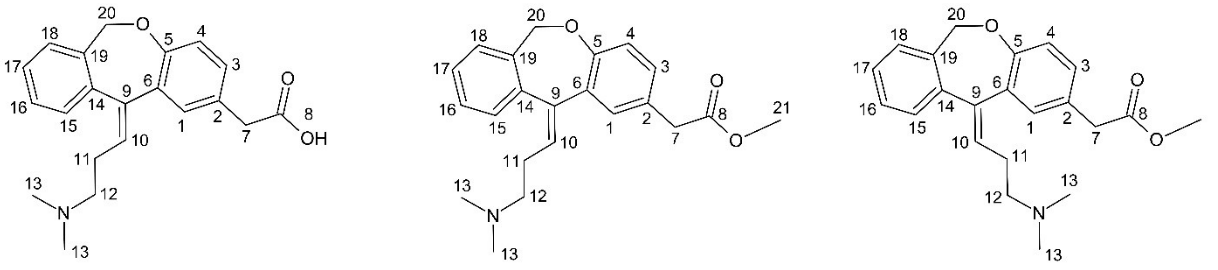

4 5 6

Figure S20. The structures of OLO4, OLO5 and OLO6.
